# Immunologic and vascular biomarkers of mortality in critical COVID-19 in a South African cohort

**DOI:** 10.3389/fimmu.2023.1219097

**Published:** 2023-07-03

**Authors:** Jane Alexandra Shaw, Maynard Meiring, Candice Snyders, Frans Everson, Lovemore Nyasha Sigwadhi, Veranyay Ngah, Gerard Tromp, Brian Allwood, Coenraad F. N. Koegelenberg, Elvis M. Irusen, Usha Lalla, Nicola Baines, Annalise E. Zemlin, Rajiv T. Erasmus, Zivanai C. Chapanduka, Tandi E. Matsha, Gerhard Walzl, Hans Strijdom, Nelita du Plessis, Alimuddin Zumla, Novel Chegou, Stephanus T. Malherbe, Peter S. Nyasulu

**Affiliations:** ^1^ Department of Science and Technology/National Research Foundation (DST-NRF) Centre of Excellence for Biomedical Tuberculosis Research, South African Medical Research Council Centre for Tuberculosis Research, Biomedical Research Institute, Division of Molecular Biology and Human Genetics, Faculty of Medicine and Health Sciences, Stellenbosch University, Cape Town, South Africa; ^2^ Centre for Cardiometabolic Research in Africa, Division of Medical Physiology, Faculty of Medicine and Health Sciences, Stellenbosch University, Cape Town, South Africa; ^3^ Division of Epidemiology and Biostatistics, Department of Global Health, Faculty of Medicine and Health Sciences, Stellenbosch University, Cape Town, South Africa; ^4^ South African Tuberculosis Bioinformatics Initiative, Stellenbosch University, Cape Town, South Africa; ^5^ Centre for Bioinformatics and Computational Biology, Stellenbosch University, Stellenbosch, South Africa; ^6^ Division of Pulmonology, Department of Medicine, Stellenbosch University and Tygerberg Hospital, Cape Town, South Africa; ^7^ Division of Chemical Pathology, Department of Pathology, Faculty of Medicine and Health Sciences, Stellenbosch University and National Health Laboratory Service, Tygerberg Hospital, Cape Town, South Africa; ^8^ Division of Haematological Pathology, Department of Pathology, Faculty of Medicine and Health Sciences, Stellenbosch University and National Health Laboratory Service (NHLS) Tygerberg Hospital, Cape Town, South Africa; ^9^ Sefako Makgatho University of Health Sciences, Ga-Rankuwa, South Africa; ^10^ Division of Infection and Immunity, Centre for Clinical Microbiology, University College London, London, United Kingdom; ^11^ National Institute for Health Care Research (NIHR) Biomedical Research Centre, University College London (UCL) Hospitals National Health Service (NHS) Foundation Trust, London, United Kingdom; ^12^ Division of Epidemiology and Biostatistics, School of Public Health, Faculty of Health Sciences, University of the Witwatersrand, Johannesburg, South Africa

**Keywords:** biomarkers, cytokines, COVID-19, SARS-CoV-2, prognostic, mortality

## Abstract

**Introduction:**

Biomarkers predicting mortality among critical Coronavirus disease 2019 (COVID-19) patients provide insight into the underlying pathophysiology of fatal disease and assist with triaging of cases in overburdened settings. However, data describing these biomarkers in Sub-Saharan African populations are sparse.

**Methods:**

We collected serum samples and corresponding clinical data from 87 patients with critical COVID-19 on day 1 of admission to the intensive care unit (ICU) of a tertiary hospital in Cape Town, South Africa, during the second wave of the COVID-19 pandemic. A second sample from the same patients was collected on day 7 of ICU admission. Patients were followed up until in-hospital death or hospital discharge. A custom-designed 52 biomarker panel was performed on the Luminex® platform. Data were analyzed for any association between biomarkers and mortality based on pre-determined functional groups, and individual analytes.

**Results:**

Of 87 patients, 55 (63.2%) died and 32 (36.8%) survived. We found a dysregulated cytokine response in patients who died, with elevated levels of type-1 and type-2 cytokines, chemokines, and acute phase reactants, as well as reduced levels of regulatory T cell cytokines. Interleukin (IL)-15 and IL-18 were elevated in those who died, and levels reduced over time in those who survived. Procalcitonin (PCT), C-reactive protein, Endothelin-1 and vascular cell adhesion molecule-1 were elevated in those who died.

**Discussion:**

These results show the pattern of dysregulation in critical COVID-19 in a Sub-Saharan African cohort. They suggest that fatal COVID-19 involved excessive activation of cytotoxic cells and the NLRP3 (nucleotide-binding domain, leucine-rich–containing family, pyrin domain–containing-3) inflammasome. Furthermore, superinfection and endothelial dysfunction with thrombosis might have contributed to mortality. HIV infection did not affect the outcome. A clinically relevant biosignature including PCT, pH and lymphocyte percentage on differential count, had an 84.8% sensitivity for mortality, and outperformed the Luminex-derived biosignature.

## 1 Introduction

Understanding the mechanisms driving severe disease and causing mortality or recovery among Coronavirus disease 2019 (COVID-19) patients is crucial for developing future clinical decision support systems, planning disease control strategies, and developing host-directed therapies. Older age, obesity, diabetes, hypertension, lymphopenia, and neutrophilia have been consistently identified in many studies as predictors of mortality (1, 2). HIV has also been associated with poor outcomes from COVID-19 in multi-center prospective studies, especially in people with a low CD4 cell count (3, 4). Many immunological biomarkers of mortality have been reported, such as interleukin-1α (IL-1α), IL-1β, IL-6, IL-10, IL-18, and tumor necrosis factor (TNF) (5, 6). Along with ferritin, D-dimer, C-reactive protein (CRP), and procalcitonin (PCT), these immunologic biomarkers are found in the group of people infected with Severe Acute Respiratory Syndrome coronavirus-2 (SARS-CoV-2) who develop hyperinflammation, known as a ‘cytokine storm’ (2). Excessive cytokine production from inflammatory cell death is associated with acute organ damage which is life-threatening. This is mediated in part by synergism between TNF and interferon gamma (IFNγ), which triggers the PANoptosis (pyroptosis, apoptosis, and necroptosis), a mechanistic compendium of programmed cell death pathways (7). In addition to hyperinflammation, endothelial markers such as intercellular adhesion molecule-1 (ICAM-1), vascular cell adhesion molecule-1 (VCAM-1), and E-selectin have been associated with disease severity and death. This highlights the important role of vascular endothelial cells as targets of SARS-CoV-2, and of endothelial activation with dysfunction in the pathogenesis of severe COVID-19 (8, 9).

Despite the extraordinary number of publications on this topic during the pandemic, few data from African countries are available in the public domain. Furthermore, Sub-Saharan African populations have a low vaccination coverage to mitigate the effects of future waves or variants which escape immunity acquired from previous infection (10). Therefore, data on the predictors of mortality in Sub-Saharan African populations are greatly needed. The second wave of COVID-19 in South Africa, which occurred between October 2020 and February 2021, was dominated by the B.1.351 Beta variant of SARS-CoV-2 (10). The rapid spread of infection in the population resulted in a high rate of admission of critically ill patients that overwhelmed the health care services. The B.1.351 Beta variant was associated with a high mortality rate among patients admitted in the Intensive Care Unit (ICU) and other healthcare environments (10).

In this study, our team examined the serum of critical COVID-19 patients in Cape Town, South Africa, for key immunological and endothelial cell biomarkers of mortality. The aim was to gain insight into the immune mechanisms underlying fatal COVID-19 and identify the biomarkers with the best predictive potential.

## 2 Materials and methods

This investigation was a sub-study of a large prospective cohort spanning the whole COVID-19 pandemic, performed in the ICU of Tygerberg Hospital, Cape Town, South Africa. Participants for this sub-study were admitted during the second wave of the COVID-19 pandemic from 9 October 2020 to 10 February 2021. All adult patients admitted to the ICU during this timeframe with laboratory-confirmed SARS-CoV-2 infection on quantitative real-time reverse transcription polymerase chain reaction (RT-qPCR) from nasopharyngeal swab testing, and COVID-19 acute respiratory distress syndrome (ARDS) according to the Berlin definition (11), were recruited on the day of admission to ICU. They were followed until the primary endpoint of death or survival to hospital discharge. Eligibility for ICU admission was predetermined by the provincial Department of Health guidelines, based on the severity of illness, likely prognosis, and ICU bed availability (12).

Baseline demographic characteristics, comorbidities, medication history, and indicators of the severity of illness (i.e., type and intensity of respiratory support, arterial blood gas values, and evidence of other organ dysfunction) were collected and transferred by authorized study staff to an access-controlled Research Electronic Data Capture (REDCap®) database hosted by Stellenbosch University (13). Baseline laboratory measurements were retrieved from the National Health Laboratory Service (NHLS) Laboratory Information System (TrakCare® Lab Enterprise). Only results from blood taken on the day of ICU admission were used. In cases where a full panel blood test was not performed on the day of ICU admission, the results from blood taken within 48 hours of the date and time of ICU admission were used. The first arterial blood gas performed after admission to ICU was selected for the analysis, irrespective of the type of respiratory support at that time. Further data on the patient’s clinical progress during admission were collected, including the primary outcome of death or discharge from the hospital, progression to mechanical ventilation, and initiation of new medications. Data were verified remotely using electronic hospital records and the TrakCare® system. Serum samples were collected from all patients on day 1 (the day of ICU admission), and a second sample was collected from those who survived to day 7, to capture the trajectory of key analytes at a time point when they were likely to differentiate between survivors and non-survivors (5).

### 2.1 Laboratory procedures

The baseline blood tests and SARS-CoV-2 RT-qPCR were performed according to the protocols of the NHLS at Tygerberg Hospital, accredited as ISO15189 compliant by the South African National Accreditation Services (SANAS), and all methods are subjected to both internal and external quality control schemes. These methods have been described in a previous publication (14).

Day 1 and day 7 serum samples were aliquoted and frozen at -80°C on the day of sampling. After a single freeze-thaw cycle, a magnetic Luminex® assay with a Luminex® MAGPIX® CCD Imager [xPONENT® software; Research and Diagnostics Systems Inc.® a Bio-techne® brand (Catalog number LXSAHM); Minneapolis, NE, USA] was used to determine the levels of a custom-designed 52-analyte panel of immune and vascular/endothelial cell biomarkers including: Arginase-1, CRP, D-dimer, Endothelin-1 (ET-1), E-Selectin, ferritin, growth differentiation factor-15 (GDF-15), granulocyte macrophage colony stimulating factor (GMCSF), granulysin, hypoxia inducible factor 1-α (HIF1α), High mobility group box 1 (HMGB1), I-309/chemokine ligand 1(I-309/CCL1), ICAM-1, Indoleamine-2,3-dioxygenase 1 (IDO-1), IFNβ, IFNγ, IL-1a, IL-1b, IL-1Ra, IL-2, IL-4, IL-5, IL-6, IL-8, IL-10, IL-13, IL-15, IL-17, IL-18, IL-21, IL-22, IL-23, IL-33, Interferon gamma-induced protein 10/Chemokine (C-X-C motif) ligand 10 (IP-10/CXCL10), monocyte chemoattractant protein 1/chemokine (C-C motif) ligand 2 (MCP-1/CCL2), MCP-3/CCL7), MCP-4/CCL13, monokine induced by gamma interferon/Chemokine (C-X-C motif) ligand 9 (MIG/CXCL9), myeloperoxidase (MPO), plasminogen activator inhibitor-1 (PAI-1), PCT, P-selectin, S100 calcium-binding protein A8/migration inhibitory factor-related protein 8 (S100A8/MRP-8), S100 calcium-binding protein A9/migration inhibitory factor-related protein 14 (S100A9/MRP-14), growth stimulation gene-2/Interleukin-1 receptor-like-1 (ST2/IL-1RL1), transforming growth factor β1 (TGFβ1), TGFβ2, TGFβ3, TNFα, VCAM-1, vascular endothelial growth factor (VEGF), Von Willebrand Factor A2 (vWF A2). Analyte kits were supplied by Whitehead Scientific (Pty) Ltd, Cape Town, South Africa, and Merck (Pty) Ltd, Gauteng, South Africa.

### 2.2 Statistical analysis

Data were analyzed using R suite® statistical software (R version 4.2.3) (15). Selected clinical variables, NHLS-derived laboratory variables, and all Luminex®-derived variables were assessed for their effect on the primary outcome of death, or survival to hospital discharge. Pairwise comparisons were done using robust t-tests for the continuous variables, and Cochran-Armitage tests for the trends of the categorical variables. Analytes where all measured values were lower than the limit of detection were excluded from this analysis. For patients with values on both days 1 and 7, a paired Yuen’s t-test with standardized winsorization was used to assess the effect of both values on the outcome. Analytes for which most of the results were lower than the limit of detection but had at least five valid entries (IL-1a, IL-4, IL-5, IL-21, IL-22, IL-23, and HMGB-1) were converted into categorical variables for the specific sample day using the minimum, maximum, and median of the measurable values, as shown in **Supplementary Table 1**. The minimum value indicated the lowest concentration (including those extrapolated downwards), while the maximum value was the highest concentration and any values which were extrapolated upwards, and the median was simply the standard median value. The analytes were then stratified by the outcome status of death or survival, and a Cochran-Armitage test for trend was applied.

Variables with the highest impact on the outcome from day 1 and day 7 separately, as well as the difference between day 1 and day 7 values in patients who had both day 1 and day 7 values measured (hereafter the trajectory analysis), were identified using a Boruta algorithm. Boruta is an all-relevant feature-selection algorithm that makes use of a permuted random forest selection process. It was chosen for this analysis because of the rigorous method by which it identifies variables with a real impact on the outcome, without relying on assumptions about the distribution of the data or excluding all variables with incomplete data in the way that other tools would. Through multiple iterations, it provides a ranking of variables’ importance to the outcome as well as a statistical interpretation of the performance based on the binomial distribution, i.e., a score of the feature importance (16). On day 1 analysis, all Luminex® analytes and the baseline clinical variables were included in the Boruta. On the day 7 analysis, and the trajectory analysis only the Luminex® analytes were included. The variables thus identified were further subjected to correlation-based filtering, and the remaining variables were then used as predictors in two independent classification models (logistic regression and a random forest model). V-fold cross-validation (stratified by outcome, with five repeats) was applied to obtain measures of performance. A second analysis was done using predetermined ‘functional groups’ of cytokines and vascular/endothelial biomarkers (**Table 1**). These were analyzed together to assess their combined effect, using a robust t-test on a pooled average value for that group, obtained by scaling and centering each analyte separately (independent of the outcome state), then calculating the mean for each patient. A p < 0.05 was considered significant. However, to minimize the impact of multiple testing and forgo *post-hoc* correction, those interactions which remained significant at p < 0.005 are highlighted.

**Table 1:** List of predetermined functional groups of analytes.

Group number	Group name	Analytes
1	Clinical markers of inflammation or infection	CRP; Ferritin; PCT; D-dimer
2	Th1	IFNγ, TNFα, IL-2, IL-12, IL-15
3	Th2	IL-4, IL-13, IL-5, IL-33, ST2, IL-21
4	Treg	IL-10, TGFβ
5	Th17	IL-17, IL-22, IL-23
6	Th1 activation	TNFα; IL-6, IL-1b, IL-1a, IL-18; IL-15
7	Th2 activation	IL-4; IL-13; IL-5; IL-10
8	Myeloid derived suppressor cells	Arginase-1; S100A8; S100A9; TFGβ; IL-10; IDO-1; IL-1Ra
9	Anti-inflammatory myeloid cells	IL-1Ra
10	Antiviral	IFNβ
11	Chemokines	IL-8; MCP-1, MCP-3, MCP-4, MIG, IP-10; i309
12	Vascular/endothelial adhesion molecules	VCAM-1, ICAM-1
13	Growth factors	GMCSF; VEGF
14	NK and CTL cytolytic activity	Granulysin
15*	Expanded vascular/endothelial adhesion markers	ICAM-1, E-selectin, P-selectin, VCAM-1
16*	Inflammation	MPO, TNFα, CRP
17*	Hemostatic factors	PAI-1, vWFA2
18*	Cell growth/death factors	GDF-15, VEGF
19*	Vascular tone	Endothelin-1

*These groups were designed to focus on markers of relevance to the vascular endothelium.

CRP, C-reactive protein; CTL, cytotoxic T lymphocyte GDF-15, growth differentiation factor-15; GM-CSF, granulocyte macrophage colony stimulating factor; I-309, chemokine ligand 1(CCL1); ICAM-1, intercellular adhesion molecule-1; IDO-1, Indoleamine-2,3-dioxygenase 1; IFNβ, interferon β; IFNγ, interferon γ; IL, interleukin; IL-1Ra, Interleukin-1 receptor antagonist; IP-10, interferon γ-induced protein 10/Chemokine (C-X-C motif) ligand 10 (CXCL10); MCP, monocyte chemoattractant protein; MIG, monokine induced by interferon γ/Chemokine (C-X-C motif) ligand 9 (CXCL9); MPO, myeloperoxidase; NK, natural killer cell PAI-1, plasminogen activator inhibitor-1; PCT, procalcitonin; S100A8, S100 calcium-binding protein A8/migration inhibitory factor-related protein 8 (MRP-8); S100A9, S100 calcium-binding protein A9/MRP-14; ST2, growth stimulation gene-2/Interleukin-1 receptor-like-1 (IL-1RL1); TGFβ, transforming growth factor β; TNFα, tumour necrosis factor α; VCAM-1, vascular cell adhesion molecule-1; VEGF, vascular endothelial growth factor; vWF A2, Von Willebrand Factor A2.

## 3 Results

**Table 2** presents the baseline characteristics of the 87 critically ill COVID-19 patients included in the analysis, of whom 55 (63.2%) died in ICU, and 32 (36.8%) survived to hospital discharge (median 3 days after ICU discharge, range 1–48 days). All patients were receiving 8mg of intravenous dexamethasone daily, and therapeutic dose subcutaneous enoxaparin sodium from admission to ICU. None of the patients received any antiviral treatment or immunomodulatory drugs for COVID-19.

**Table 2:** Baseline demographic characteristics and laboratory parameters of the study population.

	Pooled (n=87)	Died (n=55)	Survived (n=32)	P value^+^
Female	58 (66.7%)	36 (65.5%)	22 (68.8%)	0.817
Age (years)	55.0 (15.5)	55.0 (14.5)	51.5 (18.5)	0.550
Length of hospital stay (days)	12.0 (9.0)	11.0 (8.5)	13.5 (10.2)	–
Length of ICU stay (days)	10 (8.0)	10 (8.0)	9 (8.0)	–
Type of respiratory support on day of ICU admission: Invasive mechanical ventilation Non-invasive mechanical ventilation High-flow nasal oxygen Face mask oxygen Required invasive mechanical ventilation during ICU admission	32 (36.8%) 4 (4.6%) 50 (57.5%) 1 (1.1%) 59 (67.8%)	31 (56.4%) 4 (7.3%) 20 (36.4%) 0 (0.0%) 54 (98.2%)	1 (3.1%) 0 (0.0%) 30 (93.8%) 1 (3.1%) 5 (15.6%)	- - - - -
Comorbidities: Diabetes mellitus HbA1c >6.5% in ICU^$^ Hypertension HIV infection Raised BMI^€^	19 (21.8%) 49 (56.3%) 51 (58.6%) 13 (15.5%) 55 (63.2%)	10 (18.2%) 33 (60.0%) 32 (58.2%) 9 (17.0%) 36 (65.5%)	9 (28.1%) 16 (50.0%) 19 (59.4%) 4 (12.9%) 19 (59.4%)	0.295 0.380 0.999 0.759 0.796
Baseline arterial blood gas^#^: pH PaCO_2_ (kPa) PaO_2_ (kPa) PaO_2_/FIO_2_ (mm Hg)* Lactate	7.4 (0.1) 5.5 (1.4) 8.0 (2.2) 75.0 (36.4) 1.6 (0.8)	7.4 (0.1) 6.0 (1.4) 8.0 (2.4) 71.0 (35.4) 1.6 (1.0)	7.5 (0.0) 5.0 (0.5) 8.2 (2.1) 84.2 (42.2) 1.4 (0.8)	**0.000** **0.001** 0.539 **0.041** 0.377
Baseline laboratory results: Creatinine (μmol/L) eGFR (mL/min) Alanine transferase (U/L) Hemoglobin (g/dL) White cell count Lymphocytes % Neutrophil % C reactive protein (mg/L) Procalcitonin (ng/mL) Troponin T (ng/L) NT-proBNP (pg/mL) Ferritin (μg/L) D-dimer (μg/mL)	76.0 (33.5) 79.0 (35.0) 37.5 (27.2) 12.5 (1.6) 11.9 (6.8) 8.2 (5.5) 86.4 (10.2) 154.0 (152.5) 0.3 (0.9) 13.0 (19.2) 189.5 (577.0) 737.0 (853.5) 0.9 (2.1)	77.0 (41.5) 76.0 (37.5) 36.0 (27.0) 12.2 (1.8) 12.8 (7.3) 6.8 (5.7) 88.7 (9.2) 162.0 (138.0) 0.3 (1.5) 15.0 (19.0) 284.0 (987.0) 718.0 (881.5) 1.1 (3.0)	76.0 (18.0) 87.0 (28.5) 41.0 (42.0) 12.7 (1.2) 10.5 (5.4) 9.8 (5.3) 84.2 (6.8) 106.5 (124.0) 0.1 (0.3) 6.0 (11.0) 111.0 (196.0) 895.0 (763.0) 0.4 (1.3)	0.593 0.201 0.183 0.129 0.052 **0.017** 0.101 **0.025** **0.027** 0.061 0.133 0.612 0.095

Categorical variables are expressed as a number followed by a percentage and the continuous variables are expressed as a median followed by the interquartile range. Laboratory values refer to those obtained on the day of admission to the ICU (day 1). ^$^Indicates an elevated glycated hemoglobin fraction, noted independent of an established diabetes diagnosis. ^€^A subjective opinion of the treating clinician rather than objective measurement, and as such should be interpreted with caution. ^#^The first arterial blood gas done on admission to ICU, whilst receiving oxygen therapy or ventilatory support. *PaO_2_/FIO_2_ is the ratio of arterial oxygen partial pressure (PaO_2_ in mmHg) to fractional inspired oxygen (FIO_2_ expressed as a fraction), where a value <300 indicates mild hypoxaemia, <200 moderate hypoxaemia, <100 severe hypoxaemia. ^+^p values not corrected for multiple testing effect. In this case, variables with a p-value < 0.005 may be considered most likely to have post-test significance using the modified one-step M-estimator. ICU, intensive care unit; HIV, human immunodeficiency virus; PaCO_2_, partial pressure of arterial carbon dioxide; PaO_2_, partial pressure of arterial oxygen; eGFR, estimated glomerular filtration rate (calculated using Chronic Kidney Disease Epidemiology Collaboration (CKD-EPI) formula uncorrected for ethnicity); NT-proBNP, N-terminal pro-brain natriuretic peptide. Bold values means P values which are statistically significant.

### 3.1 Clinical variables

On admission to ICU, all patients had clinically diagnosed ARDS with hypoxemia: 68 (78.2%) had a ratio of partial pressure of oxygen in arterial blood (PaO_2_) to the fraction of inspired oxygen (FIO_2_), hereafter PaO_2_/FIO_2_, of < 100 (severe hypoxemia); 16 (18.4%) had a PaO_2_/FIO_2_ of 100 – 200 (moderate hypoxemia); and 3 (3.4%) had a PaO_2_/FIO_2_ > 200 (mild hypoxemia) (11). Most patients (59, 67.8%) required invasive mechanical ventilation during their admission. The remaining 28 (32.2%) were supported with non-invasive ventilation or high-flow nasal cannula oxygen.

All patients had at least one comorbidity, of which hypertension (51, 58.6%) was the most common. Nineteen (21.8%) patients were known to have diabetes mellitus, but 49 (56.3%) patients had an HbA1c >6.5% on admission to the ICU (including all but one of the confirmed diabetics). There were 13 people living with HIV (PLWH), with a median CD4 cell count of 238 cells/mm^3^, and all had suppressed viral loads on antiretroviral therapy except for three who had no data available. Four (4.6%) patients had previous tuberculosis, and four had chronic obstructive pulmonary disease. Nine (10.3%) patients reported hyperlipidemia and two (2.3%) reported ischaemic heart disease, two asthma, two hypothyroidism, previous malignancy, and chronic kidney disease, respectively. Three patients (3.4%) were pregnant on admission. Variables associated with mortality are detailed in **Table 2**. In addition to these, patients receiving antibiotics at any time during their ICU stay had significantly higher odds of death (Odds ratio 10.1, 99.5% C.I. 2.9–55, p < 0.001).

### 3.2 Biomarkers

**Figure 1:**
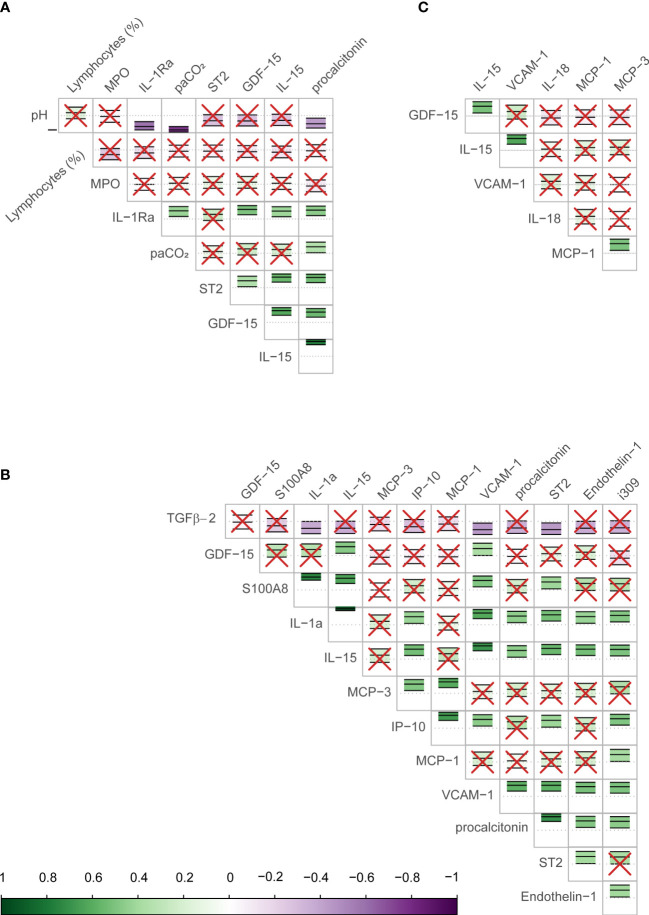
Correlation between biomarkers identified as important in predicting COVID-19 mortality in the study sample. The figure shows the correlation (Pearson) between biomarkers identified by the Boruta algorithm as important in determining the outcome (mortality) for day 1 **(A)**, day 7 **(B)**, and the longitudinal trajectory between days 1 and 7 **(C)**. The scale bar on the bottom of the figure shows the strength of the correlation (closer to 1 or -1 are strongly positive or negative respectively) with a corresponding color scale. Within each cell is a central dotted line representing 0, and the green or purple annotation represents the correlation coefficient and confidence intervals for the two biomarkers interacting in that cell, as well as the direction of the interaction. The biomarker names are shown on the labels of the rows and columns. In cells with red crosses, the confidence interval crosses the 0 line and these interactions are non-significant. Those without crosses represent the significant correlations between the biomarkers, the strength of association may be judged by their color.

The Boruta algorithm identified the following combination of clinical and Luminex® variables measured on day 1 as most influential in predicting the outcome: pH, partial pressure of carbon dioxide in arterial blood (PaCO2), lymphocytes percentage on the differential count, PCT, GDF-15, IL-15, ST2, IL-1Ra, and MPO. Only Luminex® data were considered for day 7, as all clinical data were captured on day 1. The most important day 7 variables identified by the Boruta were: IL-15, ET-1, GDF-15, IL-1a, IP-10, MCP-1, MCP-3, PCT, ST2, TGFβ2, VCAM-1, I309, and S100A8. The following variables were the most important in trajectory analysis: IL-15, GDF-15, VCAM-1, MCP-1, IL-18, and MCP-3. Several of these variables were highly correlated with each other (**Figure 1**).

**Figure 2:**
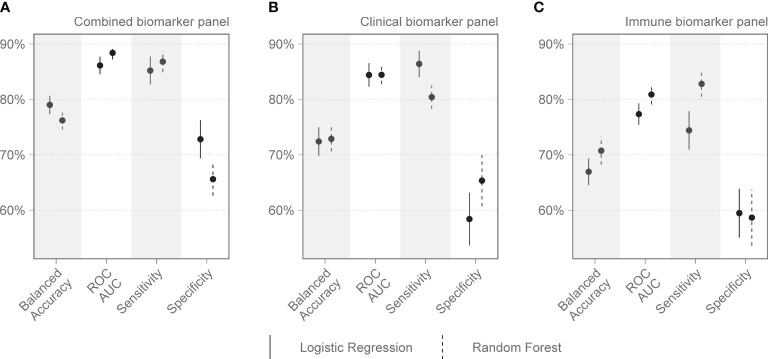
Performance metrics for models of clinical and immunologic biomarkers in predicting COVID-19 mortality on admission to the Intensive Care Unit (Day 1). **(A)** shows the combined performance of both clinical and immunologic biomarkers identified by the Boruta algorithm. **(B)** shows the performance of a clinical biomarker panel including pH, procalcitonin (PCT), and lymphocyte percentage on the differential count. **(C)** shows the performance of an immunologic biomarker panel including IL-15, MPO, GDF-15, ST-2, and IL-1Ra. Each dot represents the mean, with the line extending from it representing the standard error. A solid line is the logistic regression model, and a dashed line is the random forest model, both tuned to balanced accuracy. ROC, receiver operating curve; AUC, area under the curve.

**Figure 3:**
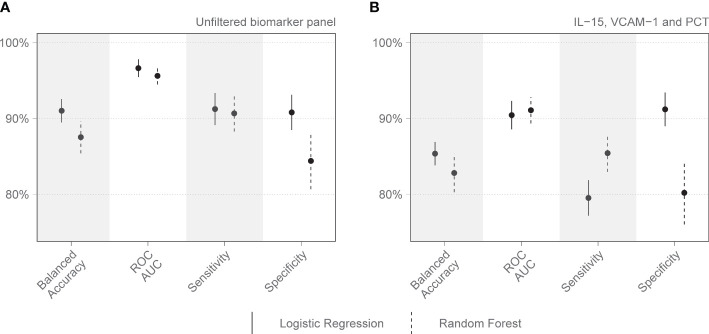
Performance metrics for models of immunologic biomarkers in predicting COVID-19 mortality on day 7 of admission to the Intensive Care Unit. **(A)** shows the performance of all day 7 analytes identified by the Boruta algorithm, without filtering. **(B)** shows the performance of the combination of analytes selected after correlation-based filtering, including IL-15, VCAM-1, and PCT. Each dot represents the mean, with the line extending from it representing the standard error. A solid line is the logistic regression model, and a dashed line is the random forest model. Models are tuned to balanced accuracy. ROC receiver operating curve, AUC area under the curve.

On day 1 two selections were tested: Group 1 included variables that are readily available in a clinical setting: pH, lymphocyte percentage on the differential count, and PCT. Group 2 included IL-15, MPO, GDF-15, ST-2, and IL-1Ra. Other combinations with randomly selected day 1 variables were also tested, as well as all variables combined. The day 1 clinical biomarker group achieved a diagnostic accuracy of 72.7% on logistic regression and 73.5% on random forest, with an area under the receiver operating curve (AUC) of 85.8% and 82.8%, respectively (**Figure 2**). Day 1 clinical biomarkers had higher sensitivity (84.8% and 82.8%) than specificity (60.7% and 64.1%). The day 1 Luminex® biomarker group achieved a diagnostic accuracy of 65.1% on logistic regression and 66.7% on random forest, with an AUC of 77.3% and 80.2% respectively. The sensitivities of this group were 78.0% and 80.4%, and specificities were 52.3% and 52.9%. When all the day 1 clinical and Luminex® biomarkers were included without filtering, the diagnostic accuracy was 78.1% and 75.1% on logistic regression and random forest models respectively; the AUCs were 86.2% and 88.6%; sensitivities were 83.2% and 88.8%; and specificities were 73.1% and 61.3%. The following day 7 variables were included in the modeling: IL-15, VCAM-1, and PCT. The performance metrics for the day 7 biomarkers were: diagnostic accuracy of 85.2% and 82.6% (logistic regression and random forest respectively); AUC of 91.1% and 93.0%; sensitivity of 80.1% and 86.5%; and specificity of 90.2% and 78.8% (**Figure 3**). When all the day 7 variables were included in the model without filtering, the metrics improved further (accuracy: 90.7% and 88.1%; AUC: 96.7% and 95.1%; sensitivity: 91.6% and 90.2%; specificity: 89.8% and 86.0%). Variables modeled from the trajectory analysis included IL-15, IL-18, and MCP-1. The biomarker trajectory analysis from day 1 to 7 performed less well than the day 7 panel, but performed well in comparison with the other models (**Supplementary Figure 1**). To test whether there was a confounding effect of HIV, we ran the same analysis excluding the 13 PLWH and compared the resultant performance metrics to the results of the whole cohort using Welch’s t-test. There was no difference between the whole cohort and the cohort with PLWH filtered out in the day 1 performance metrics, or in the day 7 metrics from the random forest models. The day 7 logistic regression metrics were better in the cohort without the PLWH, but this is likely because of the tendency of logistic regression to overfit in small datasets (**Supplementary Figure 3**).

**Figure 4:**
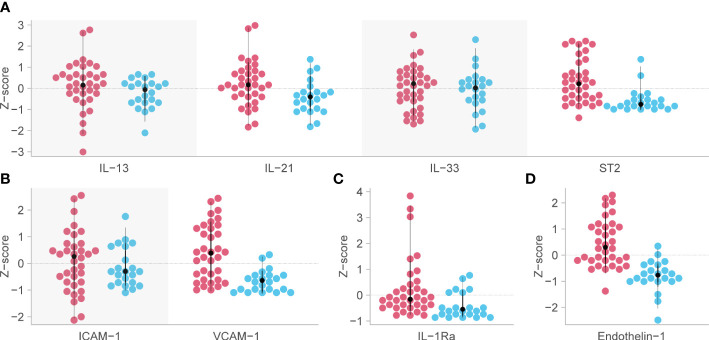
Biomarkers associated with mortality in critical COVID-19 which remained significant at the p<0.005 level. Red dots indicate the analyte levels in patients who died and blue dots are those who survived. **(A)** functional group 3, representing Th2 responses [except for Interleukin (IL)-4 and IL-5] on day 7 post-admission. **(B)** functional group 12, representing intercellular adhesion molecule-1 (ICAM-1) and vascular cell adhesion molecule-1 (VCAM-1) on day 7 post-admission. **(C)** IL-1Ra or functional group 9, representing anti-inflammatory myeloid cells on day 1. **(D)** Endothelin-1 (ET-1) or functional group 19, representing vascular tone and endothelial dysfunction on day 7 post-admission. Analyte levels have been scaled and transformed to Z-scores for comparability, and the means were compared with a robust t-test. ST2, growth stimulation gene-2.

**Figure 5:**
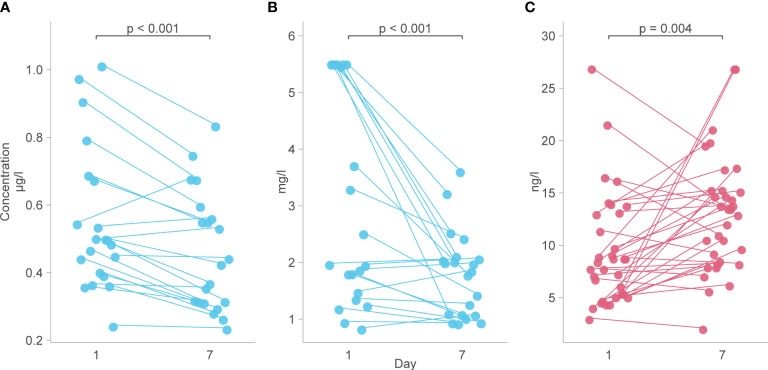
Biomarkers with a longitudinal trajectory associated with survival or death. **(A)** Levels of IL-18 between day 1 and day 7 in survivors. **(B)** Levels of VCAM-1 from day 1 to day 7 in survivors. **(C)** Levels of IL-15 from day 1 to day 7 in those who died. Analyte levels have been scaled and transformed to Z-scores for comparability and the means were compared with a robust t-test.

Analysis of predetermined functional groups identified an increase in biomarkers of a pro-inflammatory/T helper cell type 1 (Th1) activating response (IL-1α, p = 0.008; IL-1β, IL-6, IL-15, IL-18, and TNFα, p = 0.018, 95% C.I. 0.06-0.65) and the chemoattractants/leukocyte trafficking chemokines for both T helper cell type 2 (Th2) and Th1 (I309, IL-8, IP-10, MCP-1, MCP-3, MCP-4, and MIG, p = 0.034, 95% C.I. 0.02–0.57) on day 1 among patients who died. The anti-inflammatory IL-1Ra was also higher in these patients on day 1 (p = 0.003, 95% C.I. 0.04–0.45). On day 7, the inflammatory response on day 1 persisted, and Th2 cytokines (IL-13, IL-21, IL-33, and ST2, p = 0.002, 95% C.I. 0.23–0.89; IL4, p = 0.017) and acute phase pro-inflammatory markers (D-dimer, ferritin, PCT, p = 0.024, 95% C.I. 0.06–0.65) were significantly increased in those who died. Markers of an anti-inflammatory/Treg response were reduced on day 7 in those who died (IL-10, TGFβ1, and TGFβ2, p = 0.005, 95% C.I. -0.65, -0.12). Endothelin-1 (p < 0.001, 95% C.I. 0.61–1.61) and vascular endothelial adhesion markers (ICAM-1 and VCAM-1, p = 0.003, 95% C.I. 0.30–1.15) were increased in those who died. Using a 99.5% confidence interval and assuming significance at a p-value < 0.005, those interactions which remained significant included higher IL-1Ra values on day 1 in those who died (p = 0.004, 99.5% C.I. 0.00–0.52), and higher Th2 cytokines (IL-13, IL-21, IL-33, ST2, p = 0.001, 99.5% C.I. 0.08–1.04), Endothelin-1 (p <0.001, 99.5% C.I. 0.44–1.82) and ICAM-1 and VCAM-1 (p = 0.003, 99.5% C.I. 0.13–1.44) on day 7 in those who died (**Figure 4**). The results of all functional groups are shown in **Supplementary Figure 2**. The longitudinal trajectory analysis was significant for three variables: a reduction in the value of IL-18 and VCAM-1 from day 1 to day 7 was associated with survival (p = 0.002 and p = 0.004 respectively), and an increase in IL-15 from day 1 to day 7 was associated with death (p = 0.002) (**Figure 5**).

A comparison between the results of the biomarkers performed on both the Luminex® and NHLS assay platforms (CRP and PCT) is included in the **Supplementary Material** (**Supplementary Figures 4**, **5**).

## 4 Discussion

In this study, we present the immunologic and vascular biomarkers of mortality in 87 patients with critical COVID-19 who were admitted during the second wave of the pandemic in South Africa. Our results show that hyperinflammation was associated with death from COVID-19, as shown by the increased levels of Th1 and Th2 cytokines, Th1/Th2-related chemokines, and acute phase inflammatory proteins in those who died.

Cytokines related to hyperinflammation have been strongly associated with severe COVID-19 in other studies (5, 6). Similar to our findings, Lucas et al. (5) reported that patients with higher incidences of coagulopathy and mortality had a baseline immune signature driven by a combination of Th1 and Th2 responses, however, our study, unlike Lucas et al., did not identify Th17 responses as part of this signature. Moreover, Lucas et al. showed that patients with severe COVID-19 maintained elevated Th1 and Th17 cytokine levels throughout the course of their disease compared to patients with moderate disease and that severe disease was also associated with a rise in Th2 responses. In line with this, elevations in Th1 cytokines were associated with mortality at days 1 and 7 in our cohort of patients with severe COVID-19, and at the later time point, the Th2 cytokines also became significant. In Abers et al., elevated levels of IL-15, soluble ST2, and MCP-1, amongst others, were independently associated with mortality (6). Similar to our findings, the longitudinal trajectory of both IL-15 and MCP-1 in Abers et al. were significantly associated with the outcome (6). IL-1Ra is a competitive antagonist of the potent Th1 cytokine IL-1β. It rises in response to IL-1 and modulates its inductive effects on IL-6 and Th17 responses. In this study, IL-1Ra was strongly associated with mortality, providing further evidence of the presence of hyperinflammation and the body’s attempt at immune regulation (17). Many of the cytokines and chemokines reported in this study are also known to be elevated and predict mortality in other forms of ARDS and sepsis, particularly IL-6, IL-8, IL-18, TNFα, and IFNγ (18–20). However, this may not represent the cytokine milieu in the lung. In one report from Saris et al., IL-6, IP-10, MCP-1, IL-10, and IFN-α were all elevated in the plasma of critically ill COVID-19 patients (21). In the bronchoalveolar lavage fluid (BALF), however, IL-6, MCP-1, and IL-10 were significantly higher than plasma, but IP-10 was not and IFN-α was undetectable. This demonstrates that while circulating biomarkers are useful for predicting severity, they do not reveal the full picture.

IL-15 is a pro-inflammatory cytokine with important effects on the activation and cytolytic activity of cytotoxic CD8 T lymphocytes and NK cells, particularly in short-term hypoxia (22). IL-15 was significantly associated with mortality in our panel overall. IL-18 is also pro-inflammatory, activated by the NLRP3 (nucleotide-binding domain, leucine-rich–containing family, pyrin domain–containing-3) inflammasome along with IL-1β and, in combination with IL-12, acts on CD4 T cells, CD8 T cells, and NK cells to induce IFN-γ production. Higher levels of IL-18 have been associated with increased severity of COVID-19 (23). In ARDS from avian influenza virus (H5N1 and H7N9), the prolonged activation of the NLRP3 inflammasome and consequently Caspase 1, results in excess IL-18 and IL-1β production. This in turn causes an IFN-γ–biased cytokine storm, pyroptosis, and lung damage (24). In the absence of IL-15, IL-18 does not induce IFN-γ production but rather plays an important role in the differentiation of naive T cells into Th2 cells and stimulates the production of IL-4 and IL-13. In this study, we found that the survivors of COVID-19 ARDS had reduced levels of IL-15 and IL-18 over the period of ICU admission to day 7 and that the levels of these cytokines were consistently higher in those who died than those who survived. Continuous stimulation with IL-15 has been shown to cause exhaustion in certain immune cells, including NK cells (25). Together, this suggests that these pathways are involved in excessive inflammatory cell death and lung damage, and reflects the development of reduced inflammatory processes in those who survived. This may have been perpetuated by the failure of the regulatory responses which was identified at day 7 in those who died.

The chemokines as a functional group, as well as the levels and trajectory of individual chemokines such as IP-10, MCP-1, and MCP-3, featured prominently in predicting the poor outcome in our study and also shown in other studies. This highlights the role of leukocyte chemoattractants in severe disease progression (26, 27). IP-10 and MCP-1 have also been associated with thrombosis, and it is hypothesized that this may be the underlying mechanism that precipitates an increased risk of mortality (27). Both IL-15 and IP-10 (along with IL-6 and IL-10) were shown to be raised in the serum of severe COVID-19 patients as compared to severe non-COVID-19 acute respiratory illness from another cohort in the Sub-Saharan Africa region (28).

Bacterial superinfection and venous thromboembolism are known complications of critical COVID-19 (29). Our patients who died had significantly higher routine PCT values on day 1, and higher Luminex®-derived PCT, ferritin, and D-dimer values on day 7, than those who survived. We also found that receiving antibiotics during admission was associated with mortality. In addition, there was considerable overlap between the markers identified in our study and those known to be associated with mortality from bacterial sepsis (30). In combination, these findings suggest that bacterial superinfection and venous thromboembolism played a role in mortality, although neither condition was verifiable with imaging or autopsy because of the COVID-19-related restrictions and resource constraints. Theoretically, a dysregulated immune response combined with the corticosteroids that were used to treat the hyperinflammation could have resulted in an individual patient’s susceptibility to secondary infection. Bacterial superinfections occur in up to 50% of critical COVID-19 patients (29, 31). They prolong ventilation, and along with fungi are common causes of mortality in critical COVID-19 (31, 32). They generally occur later in the ICU admission than our samples were taken, but it may be that some of our patients presented late in the evolution of their disease (29, 31). An elevated D-dimer, however, may be indicative of more than a complication of critical COVID-19. Endotheliitis and a progressive endothelial thrombo-inflammatory syndrome have been suggested as the main pathological mechanism of organ injury in severe disease, rather than hyperinflammation (30, 33). This is partly because cytokine levels in COVID-19 hyperinflammation are profoundly lower than in non-COVID-19 ARDS and associated bacterial sepsis (30).

In addition to the hypothesized role of IP-10 and MCP-1 in thrombosis discussed earlier, the prominence of vascular/endothelial markers in all our analyses supports the endotheliitis theory. ET-1 is a potent vasoconstrictor released from endothelial and smooth muscle cells, which stimulates interleukin and TNFα expression in monocytes, leukocyte adherence, platelet aggregation, expression of adhesion molecules including ICAM-1 and VCAM-1, production and action of growth factors, DNA and protein synthesis, and cell cycle progression (34). It is a marker of endothelial dysfunction and is higher in patients with severe COVID-19, those with respiratory failure, and those who died in hospital (35, 36). The endothelium-derived vascular adhesion molecules ICAM-1 and VCAM-1 are markers of endothelial activation, have been reported as elevated in severe COVID-19, and are associated with death among patients admitted to the ICU (9, 26, 37). In addition to this, they are critical for the recruitment of inflammatory cells from circulation into the lungs, and in this way may be mediators of lung injury in ARDS (38). Indeed, VCAM-1 has been shown to be elevated in the BALF of patients with COVID-19 ARDS (39). In our study, ET-1, ICAM-1, and VCAM-1 were significantly associated with the outcome, particularly in the day 7 analysis and the trajectory analysis, where a reduction in the level of VCAM-1 from day 1 to 7 predicted survival. ET-1 and the adhesion molecules play a critical role in the pro-atherosclerotic pathway, and it is not clear in this study whether the derangements we observed were because of pre-existing vascular disease, a new onset COVID-19 endotheliitis, or some combination of the two. GDF-15 is a stress-responsive member of the TGFβ cytokine superfamily which is produced by many cell types including activated macrophages, cardiomyocytes, adipocytes, endothelial cells, and vascular smooth muscle cells. It increases during states of tissue injury and inflammation and has a tissue-protective role in sepsis, including the regulation of injury-mediated responses in the lungs (40). GDF-15 levels have been associated with cardiovascular risk and disease, including endothelial dysfunction and atherosclerosis (41). In this particular study, a high GDF-15 was strongly associated with COVID-19 mortality. A study by Ahmed et al. found that GDF-15 was a significant marker of disease severity that correlated with IL-6 as a predictor of ICU and hospital outcomes (42). Taken together these data suggest a prominent role for endothelial dysfunction and inflammation in the pathogenesis of fatal COVID-19, possibly in the context of pre-existing vascular disease.

Our models created a clinically relevant biosignature for predicting mortality on day 1 of admission to the ICU. It only included results which should be made available within a few hours of admission: lymphocyte percentage on the differential count, pH, and PCT. With sensitivities of 82.8% and 84.8%, this signature might be worth further investigation for use as a triaging tool in the overburdened ICU settings during pandemic times. The combination of clinical and Luminex®-derived biomarkers from day 1 performed even better, and our Luminex®-alone signature from day 7 did better on performance metrics than most other reported scoring systems. However, this finding should be regarded with caution. Our sample was from a highly selected population of critically ill COVID-19 ARDS patients from a specific geographic region. Other scoring systems may suffer the same limitations as documented in a critical appraisal of 39 prognostic models for mortality risk in COVID-19, only one model did not show a high risk of bias (1). This model, the 4C mortality score, predicted in-hospital mortality using patient age, sex, number of comorbidities, respiratory rate, peripheral oxygen saturation, level of consciousness, urea level, and CRP. The AUC was 0.79 for the derivation cohort and 0.77 for the validation cohort (43). Our clinical model for use on day 1 in the ICU has fewer variables than the 4C score and our results suggest that if there was a point-of-care assay for some of the Luminex® analytes we could provide critical care clinicians with a highly sensitive and specific prediction score which is easily available at the bedside. The biosignature we found may also be valid in ARDS of other causes considering the similarity between the biomarkers that we have shown and ARDS of other causes, and the significant association between ARDS severity (the PaO_2_/FIO_2_) and mortality.

Two of the strongest clinical markers of prognosis in this study were the pH and PaCO_2_ on arterial blood gas. COVID-19 ARDS usually presents as a Type 1 respiratory failure with severe hypoxemia, rather than Type 2 where hypercapnia is the dominant feature. In our patients who all had severe hypoxemia, a rising PaCO_2_ and drop in pH likely indicated a severe ventilation-perfusion mismatch (from either lung or vascular pathology), where both the transfer of O_2_ into the alveolar capillaries and the clearance of CO_2_ were impaired. Consistent with this, a rising PaCO_2_ trajectory has been associated with mortality from COVID-19 in mechanically ventilated patients in a large population-based cohort study (44). The complex relationship between pH and PaCO_2_ was further explored in the larger cohort, which included the patients in this sub-study of biomarkers of mortality (14).

PLWH made up 15.5% of the study population. Even though the proportion of PLWH was higher among those who died, the data was insufficient to determine an exposure-outcome relationship. In addition, there could have been inherent selection bias due to the fact that the PLWH who were selected for admission to the ICU, based on the local eligibility criteria, were virologically suppressed on ART. Epidemiologic evidence from South Africa has shown a significant association between HIV infection and in-hospital mortality among critical COVID-19 patients (3). In this study patients who were not on ART or virologically suppressed were more likely to die in hospital than their counterparts (3). The mechanism underlying this increased risk remains unknown. A histopathology study found no difference in the lungs, liver, heart, or rate of bacterial co-infection (other than *Mycobacterium tuberculosis*) of PLWH who died of COVID-19 and the HIV-uninfected COVID-19 deceased (45). Another study done within the African continent found no significant difference in serum or nasal lining fluid cytokine responses to moderate-severe COVID-19 between PLWH and HIV-uninfected counterparts (28). However, the study was not sufficiently powered to detect statistically significant differences.

Our study was limited by the small sample size which might have underpowered its ability to detect significant differences. Other key variables such as markers of cardiac dysfunction (Troponin T and NT-proBNP) and hypercoagulability (D-dimer) were seen to be higher in patients who died, but the observed differences were not statistically significant. Other data reported from South Africa have, however, shown these biomarkers of critical illness in COVID-19 in our population to be relevant (46). Selection bias also seems to have played a role in the study as there was no significant difference between groups in age or comorbidity, despite the fact that these have been reported to be among the most common factors associated with mortality. This may be because all our patients had already developed critical illness at the time of admission such that our study sample overall was older with a high rate of comorbidity and therefore a higher risk of death. Lastly, our analysis was limited by the inclusion of only two time points, which may not provide a true reflection of the trajectory over time. Biomarker levels may have fluctuated between the two measured time points or changed significantly after day 7.

In summary, this study has added much-needed data to the pool of biomarkers of severe COVID-19 ARDS in sub-Saharan African populations. We have shown that hyperinflammation, or a severely dysregulated cytokine response, is associated with mortality in the ICU. Our results also suggest that fatal COVID-19 ARDS involves excessive activation of cytotoxic cells and the NLRP3 inflammasome. Bacterial superinfection from immune dysregulation or treatment-induced suppression, and thrombosis from underlying endothelial dysfunction, likely contributed to death in these patients. Our models have made a biosignature of fatal COVID-19 on admission to the ICU which warrants further testing.

## Data availability statement

The datasets presented in this study can be found in online repositories. The names of the repository/repositories and accession number(s) can be found below: https://figshare.com/projects/COVID19_ICU_immune_and_vascular_biomarkers_of_mortality/163708.

## Ethics statement

The studies involving human participants were reviewed and approved by Stellenbosch University Health Research Ethics Committee. Written informed consent for participation was not required for this study in accordance with the national legislation and the institutional requirements.

## Author contributions

AEZ, PN, GW, HS, BA, RE, ZC, TM, AZ and NC conceived and designed the study. CS performed the assays. CK, UL, EI, NB, LS, JS, and VN collected or contributed to the data. MM and GT performed the analysis. GW, NdP, HS, AZ, BA, FE, JS, and SM informed the interpretation of the data. JS wrote the paper, which was critically reviewed and approved in the final draft by all authors. All authors contributed to the article and approved the submitted version.
