# Supplementary material for: Immunologic and vascular biomarkers of mortality in critical COVID-19 in a South African cohort
Source: Front Immunol. 2023 Jul 3;14:1219097. doi: 10.3389/fimmu.2023.1219097 (PMC10351604; doi:10.3389/fimmu.2023.1219097)
Supplement: Supplementary file 1 [file DataSheet_1.pdf]

Supplementary Material

Immunologic and vascular biomarkers of mortality in critical COVID-19 in a South African cohort

1 Supplementary Tables

Supplementary Table 1. Analytes with > 50% of results outside the limit of detection which provided statistically significant results when converted to categorical variables.

Analyte	Concentration (x)	Survived	Died	p value
IL-1a (Day 1)	x ≤ minimum	29	39	$p = 0.008$
	minimum < x ≤ median	1	6	
	median < x ≤ maximum	0	8	
IL-4 (Day 1)	x ≤ minimum	21	24	$p = 0.017$
	minimum < x ≤ median	6	13	
	median < x ≤ maximum	3	16	
IL-23 (Day 1)	x ≤ minimum	28	32	$p < 0.001$
	minimum < x ≤ median	2	9	
	median < x ≤ maximum	0	12	
IL-4 (Day 7)	x ≤ minimum	19	15	$p < 0.001$
	minimum < x ≤ median	0	7	
	median < x ≤ maximum	0	9	

Analytes for which most of the results were outside the limit of detection (IL-1a, IL-4, IL-5, IL-21, IL-22, IL-23, HMGB-1) were converted into categorical variables for the relevant day using the minimum, maximum and median values of those values which were measurable. The minimum value indicated the lowest concentration (including those extrapolated downwards), while the maximum value was the same except also including any values which were extrapolated upwards, and the median was simply the standard median value. The analytes were then stratified by the binary outcome of death or survival, and a Cochran-Armitage test for trend was done. In the table are the analytes that had significant results in this analysis. IL, interleukin.

2 Supplementary Figures

Supplementary Figure 1. Performance metrics for models of the longitudinal trajectory of immunologic biomarkers in predicting COVID-19 mortality.

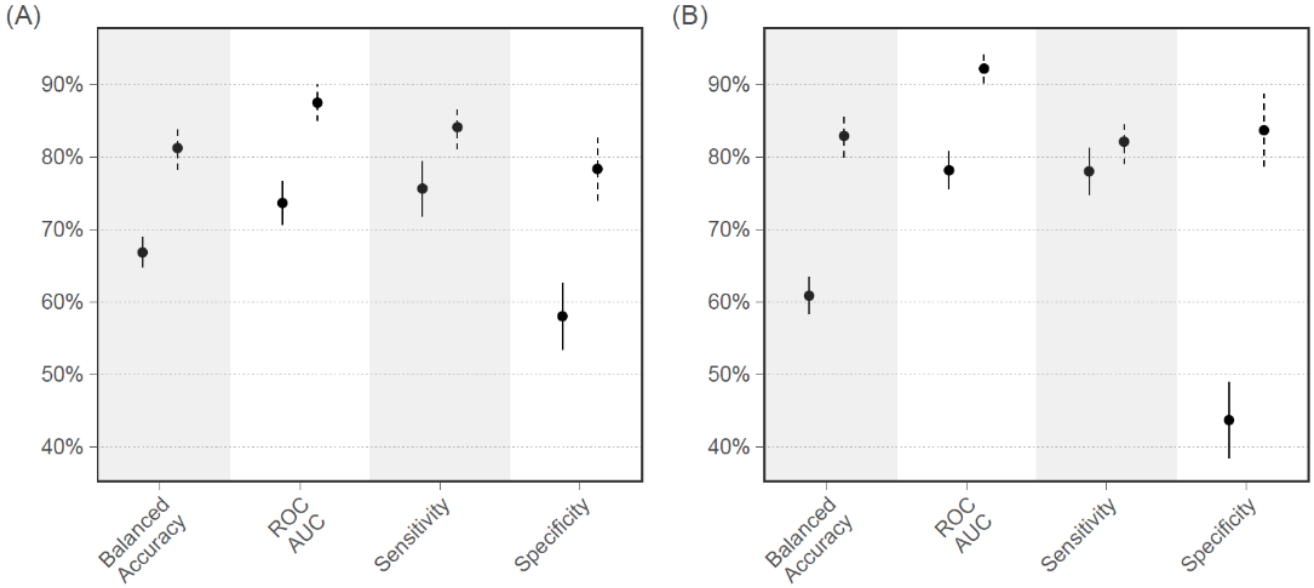

Panel (A) shows the performance of the longitudinal trajectory of all the analytes identified by the Boruta algorithm, without filtering. Panel (B) shows the performance of the combination of analytes selected after correlation-based filtering, including IL-15, IL-18, and MCP-1. Each dot represents the mean, with the line extending from it representing the standard error. A solid line is the logistic regression model, and a dashed line is the random forest model. Models are tuned to balanced accuracy. ROC AUC, receiver operating curve area under the curve.

Supplementary Figure 2. Biomarker levels in predetermined functional groups on Day 1 and Day 7 of admission to the Intensive Care Unit

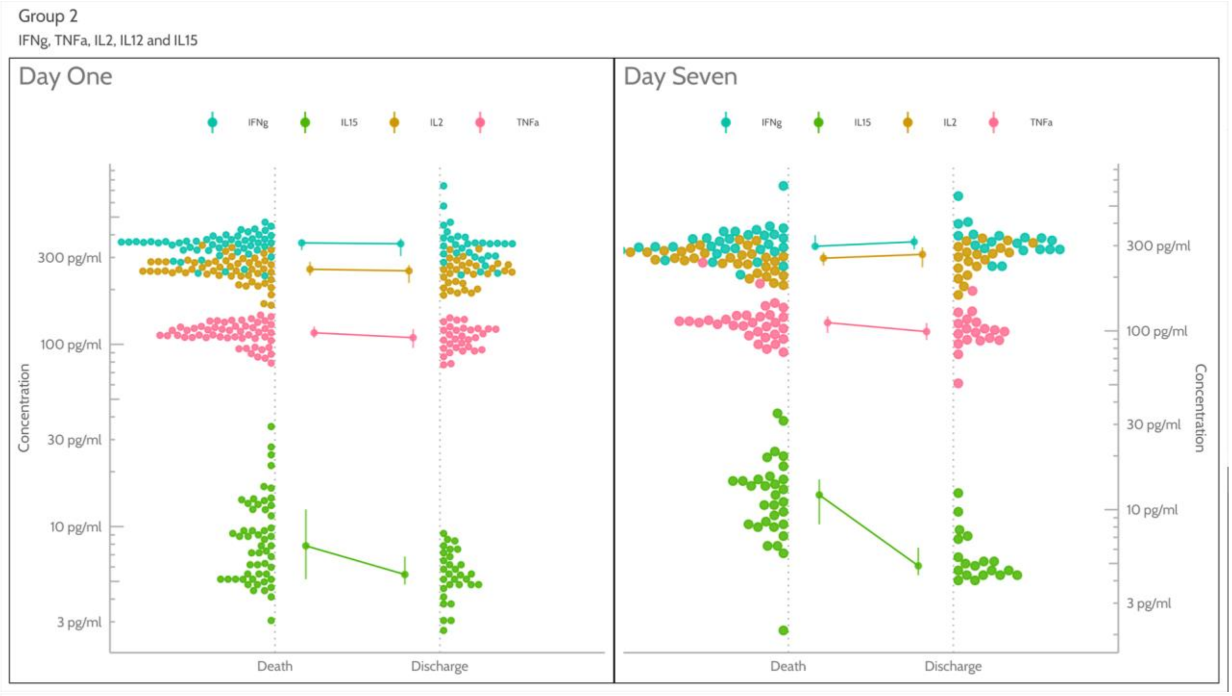

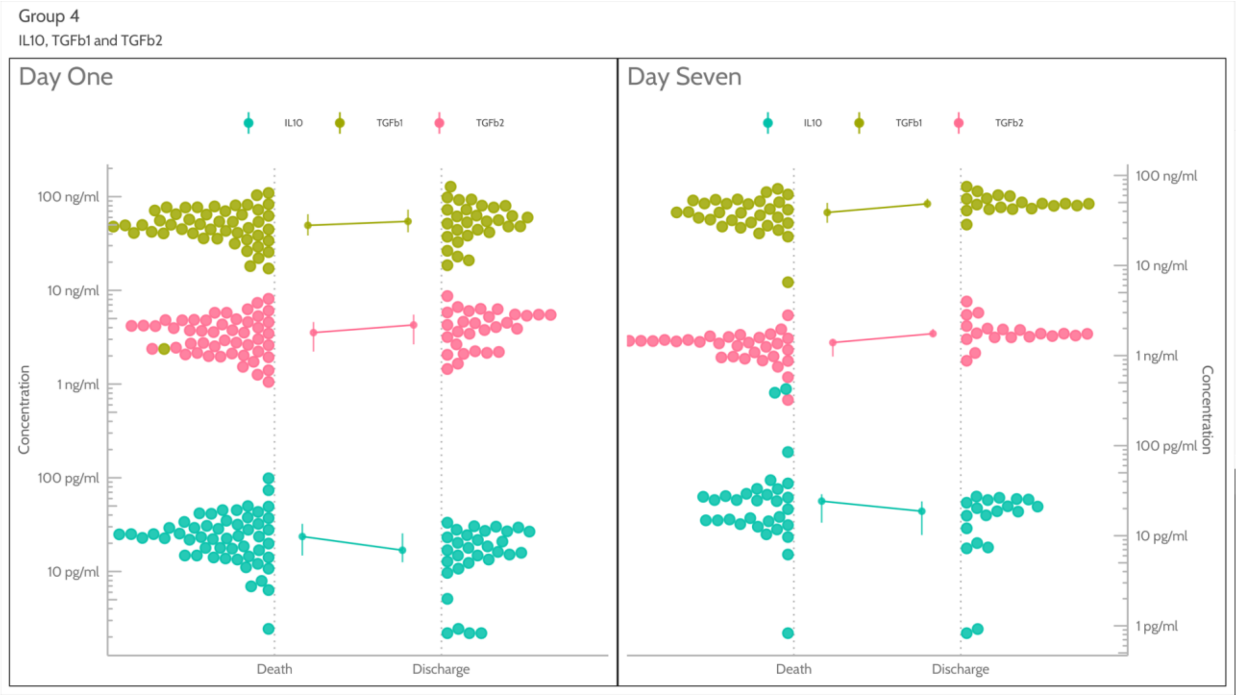

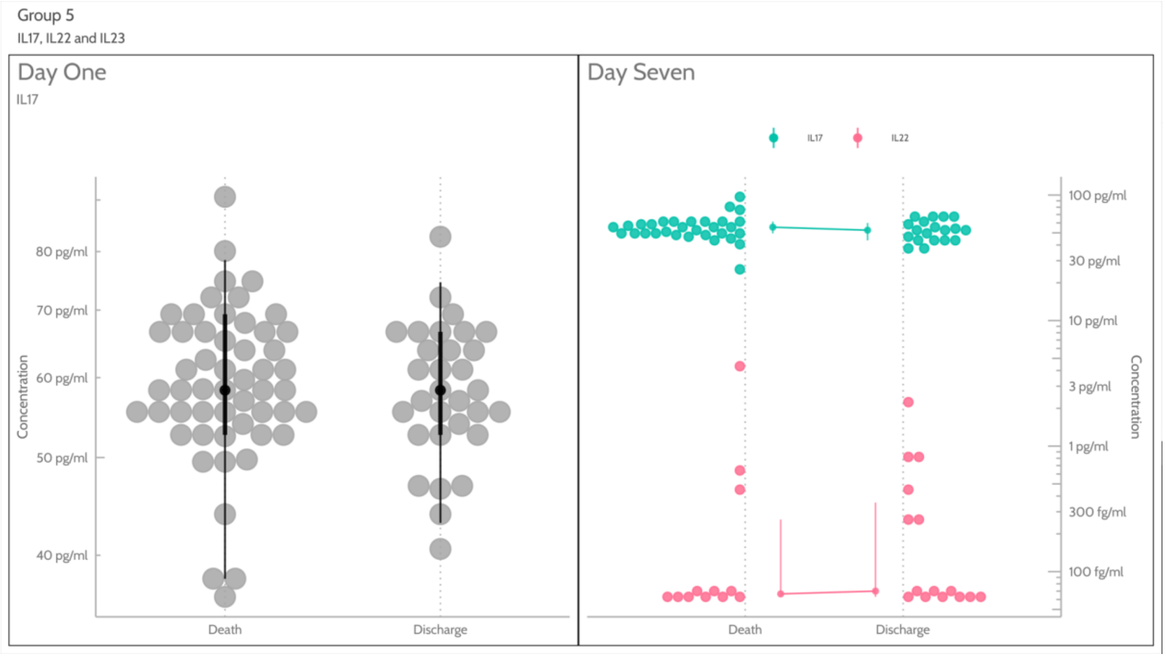
Group 5
IL17, IL22 and IL23
Day One
IL17
Concentration
80 pg/ml
70 pg/ml
60 pg/ml
50 pg/ml
40 pg/ml
Death
Discharge
Day Seven
IL17
IL22
100 pg/ml
30 pg/ml
10 pg/ml
3 pg/ml
1 pg/ml
300 fg/ml
100 fg/ml
Concentration
Death
Discharge

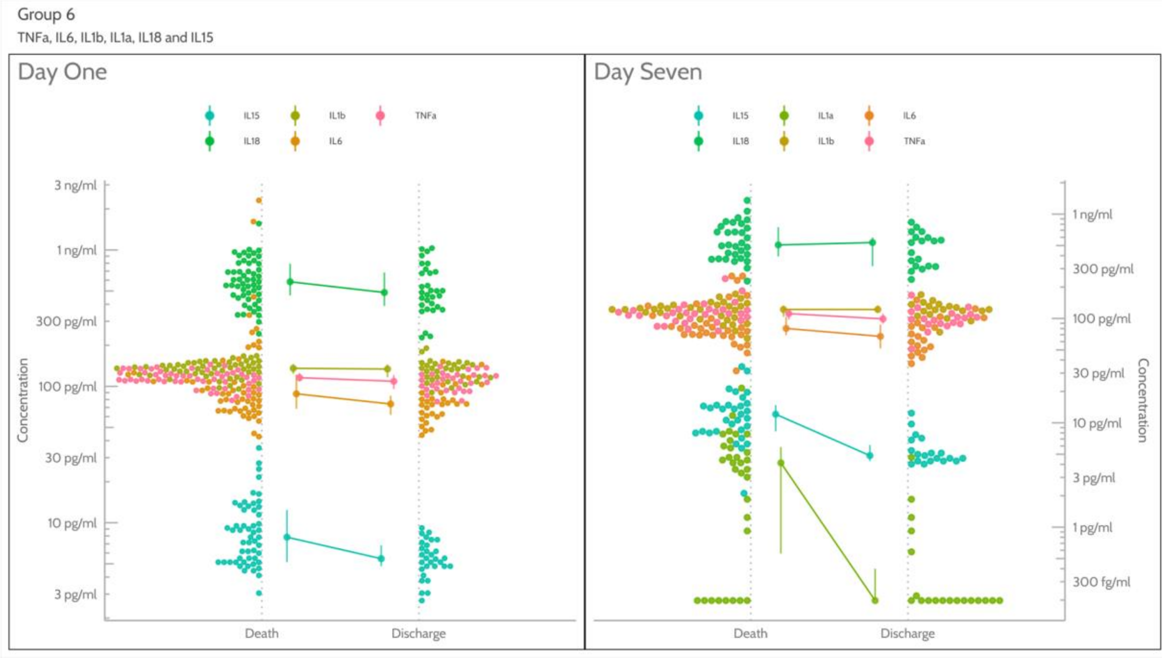
Group 6
TNFa, IL6, IL1b, IL1a, IL18 and IL15
Day One
IL15
IL1b
TNFa
IL18
IL6
3 ng/ml
1 ng/ml
300 pg/ml
100 pg/ml
30 pg/ml
10 pg/ml
3 pg/ml
Concentration
Death
Discharge
Day Seven
IL15
IL1a
IL6
IL18
IL1b
TNFa
1 ng/ml
300 pg/ml
100 pg/ml
30 pg/ml
10 pg/ml
3 pg/ml
1 pg/ml
300 fg/ml
Concentration
Death
Discharge

Group 7

IL4, IL13, IL5 and IL10

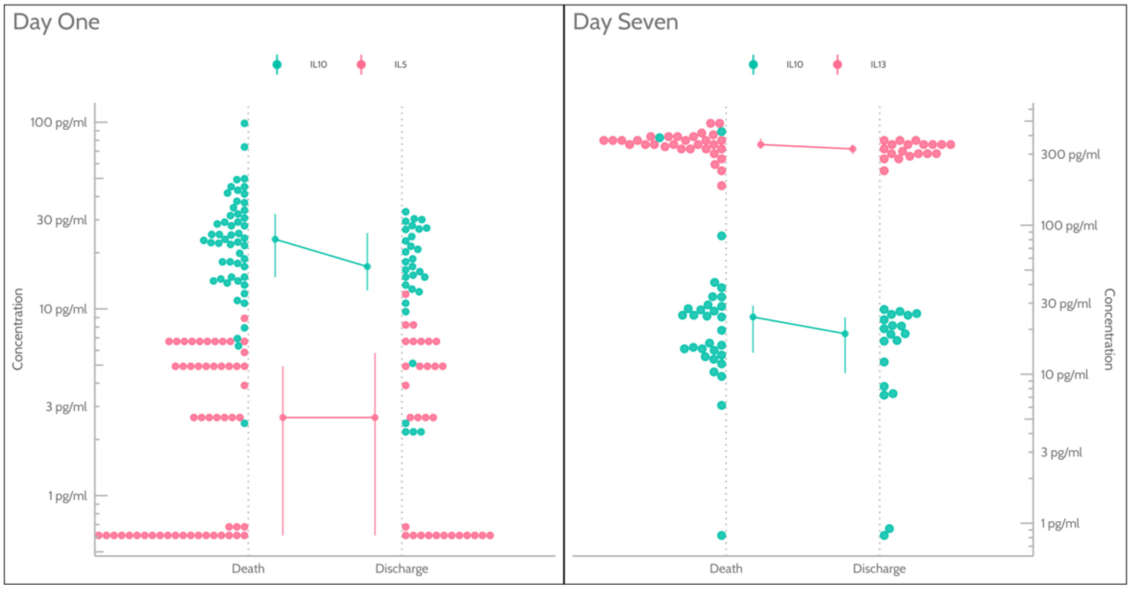

Group 8

Arginase1, S100A8, S100A9, TGFb1, TGFb2, IL10, IDO1 and IL1ra

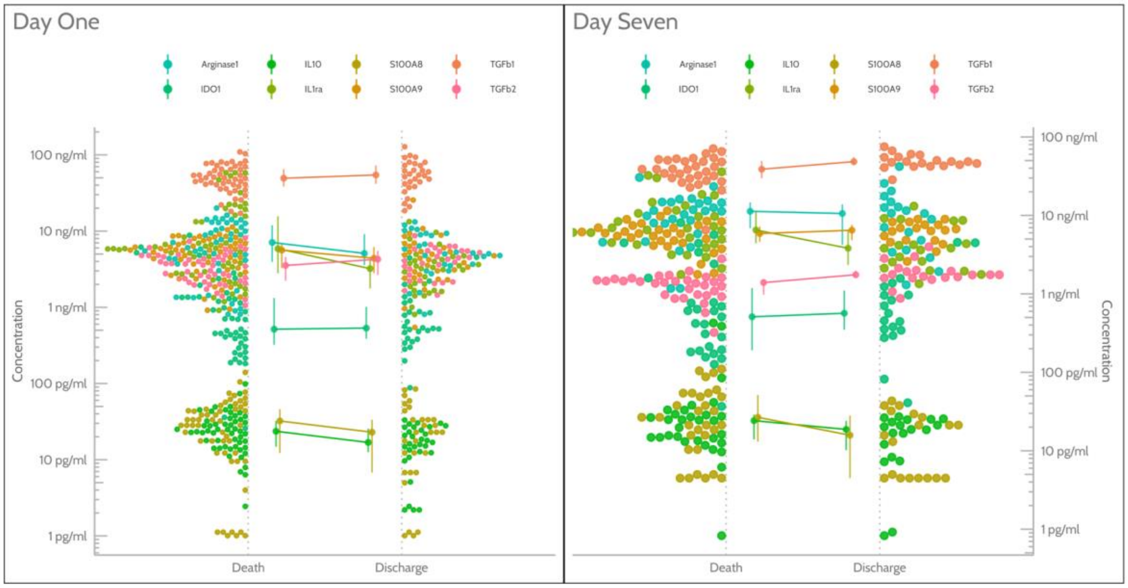

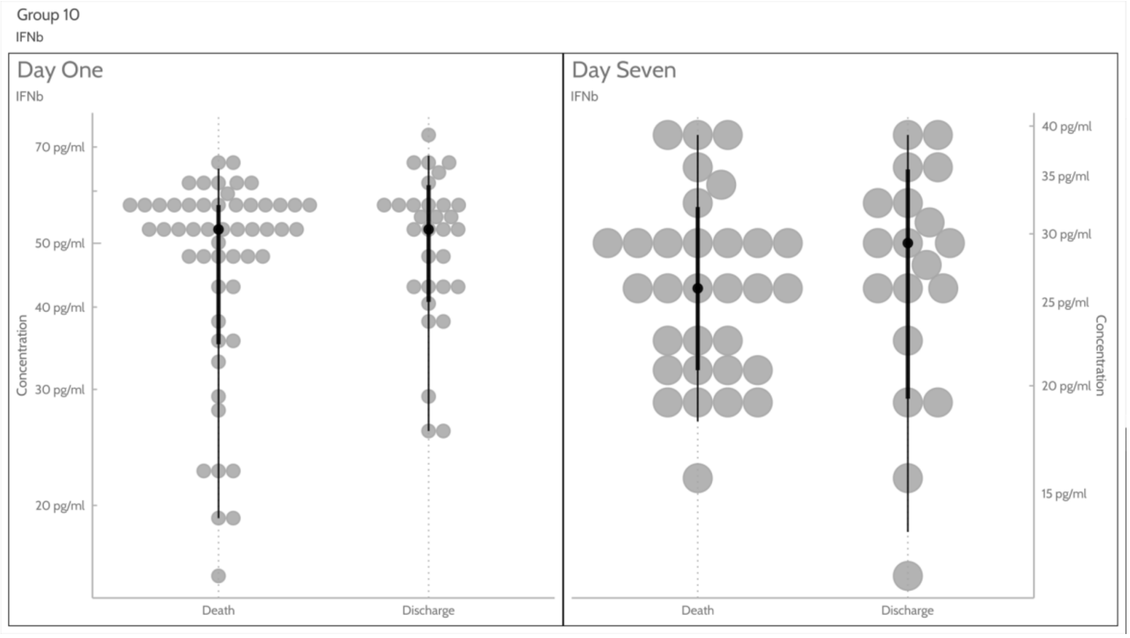

Group 10
IFNb
Day One
IFNb
70 pg/ml
50 pg/ml
40 pg/ml
30 pg/ml
20 pg/ml
Concentration
Death
Discharge
Day Seven
IFNb
40 pg/ml
35 pg/ml
30 pg/ml
25 pg/ml
20 pg/ml
15 pg/ml
Concentration
Death
Discharge

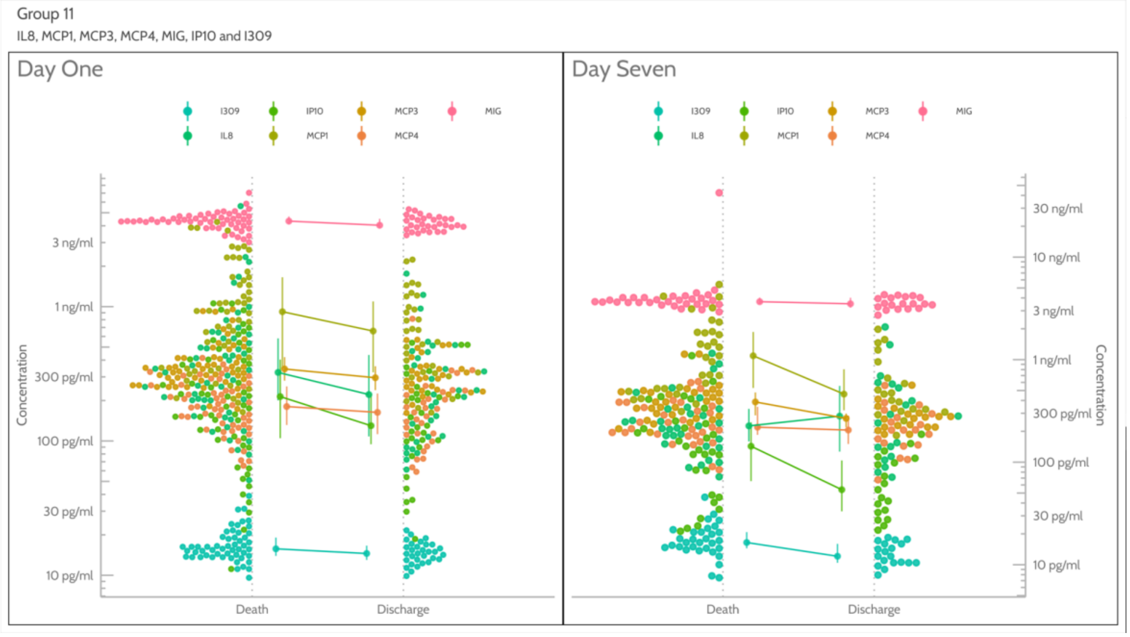

Group 11
IL8, MCP1, MCP3, MCP4, MIG, IP10 and I309
Day One
I309
IL8
IP10
MCP1
MCP3
MCP4
MIG
3 ng/ml
1 ng/ml
300 pg/ml
100 pg/ml
30 pg/ml
10 pg/ml
Concentration
Death
Discharge
Day Seven
I309
IL8
IP10
MCP1
MCP3
MCP4
MIG
30 ng/ml
10 ng/ml
3 ng/ml
1 ng/ml
300 pg/ml
100 pg/ml
30 pg/ml
10 pg/ml
Concentration
Death
Discharge

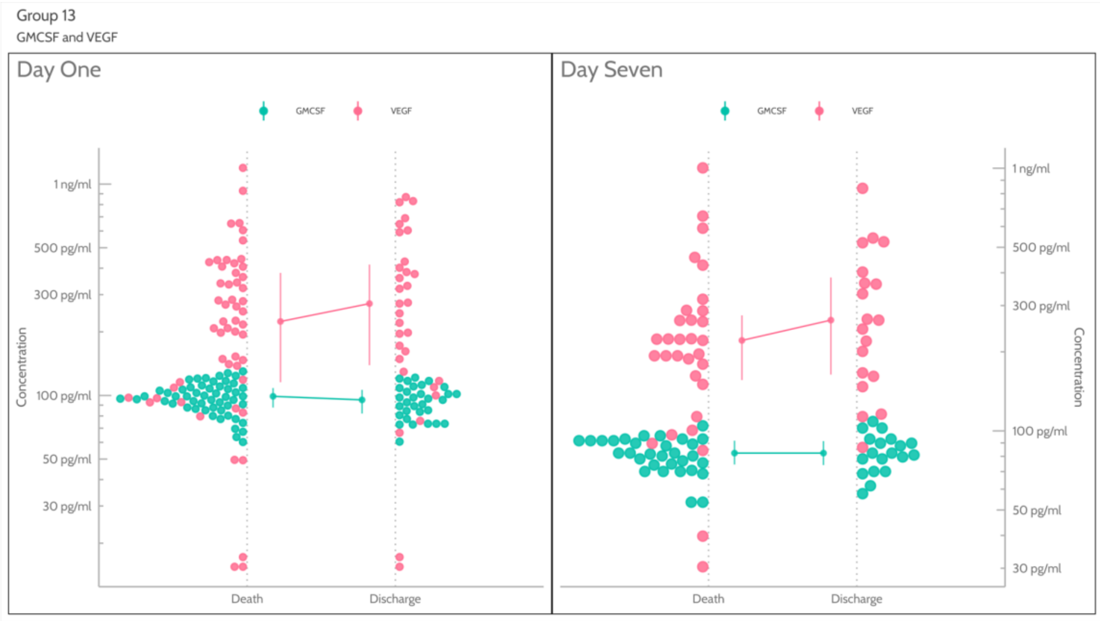
Group 13
GMCSF and VEGF
Day One
GMCSF
VEGF
1 ng/ml
500 pg/ml
300 pg/ml
100 pg/ml
50 pg/ml
30 pg/ml
Concentration
Death
Discharge
Day Seven
GMCSF
VEGF
1 ng/ml
500 pg/ml
300 pg/ml
100 pg/ml
50 pg/ml
30 pg/ml
Concentration
Death
Discharge

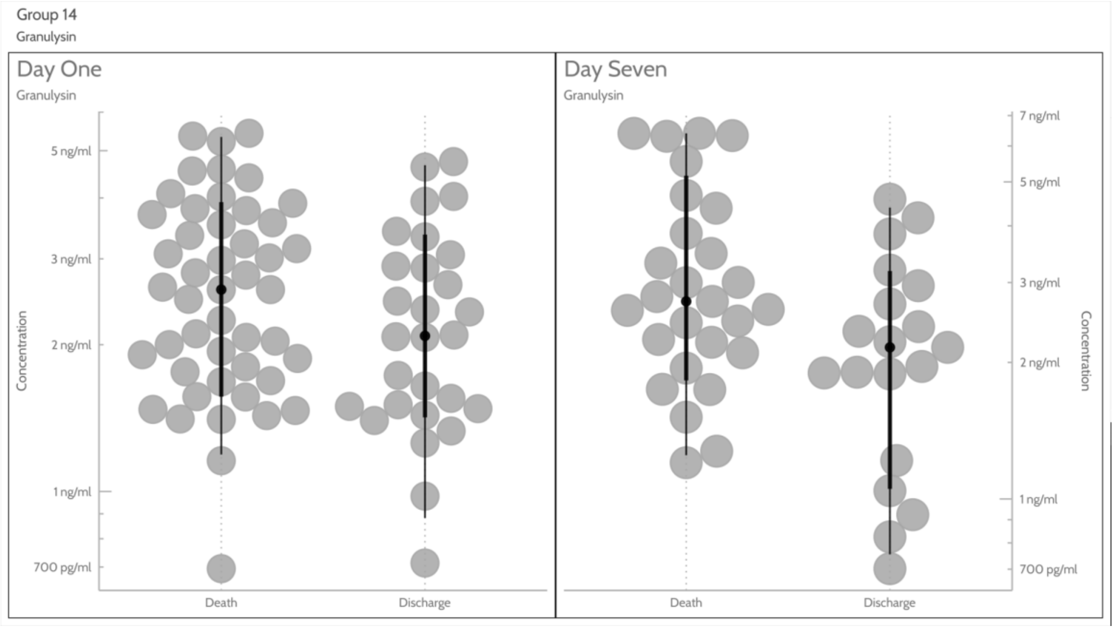
Group 14
Granulysin
Day One
Granulysin
5 ng/ml
3 ng/ml
2 ng/ml
1 ng/ml
700 pg/ml
Concentration
Death
Discharge
Day Seven
Granulysin
7 ng/ml
5 ng/ml
3 ng/ml
2 ng/ml
1 ng/ml
700 pg/ml
Concentration
Death
Discharge

Group 15
ICAM1, ESelectin, PSelectin and VCAM1

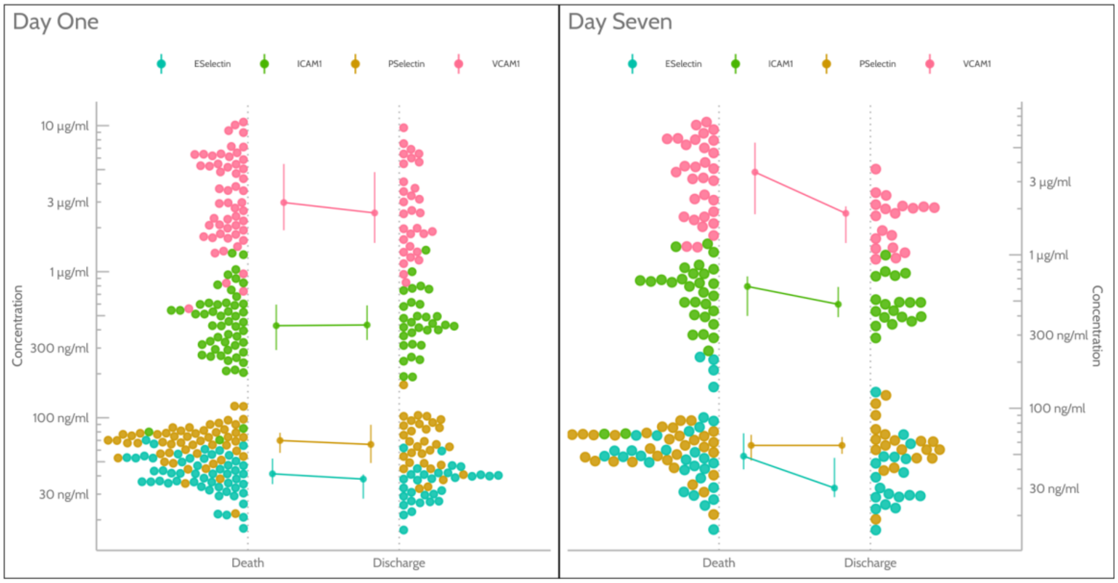

Group 16
MPO, TNFa and CRP

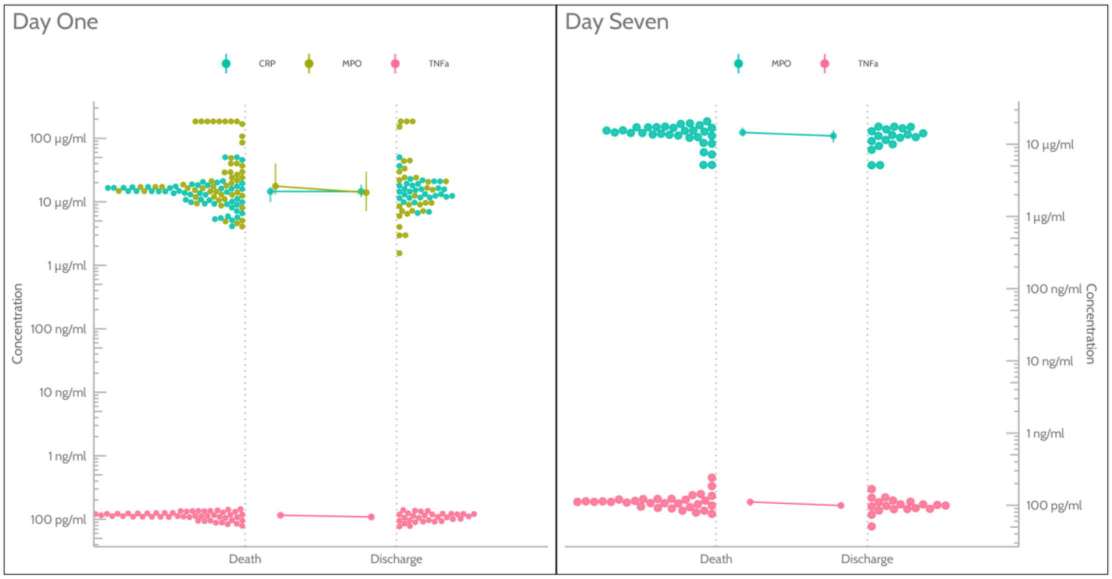

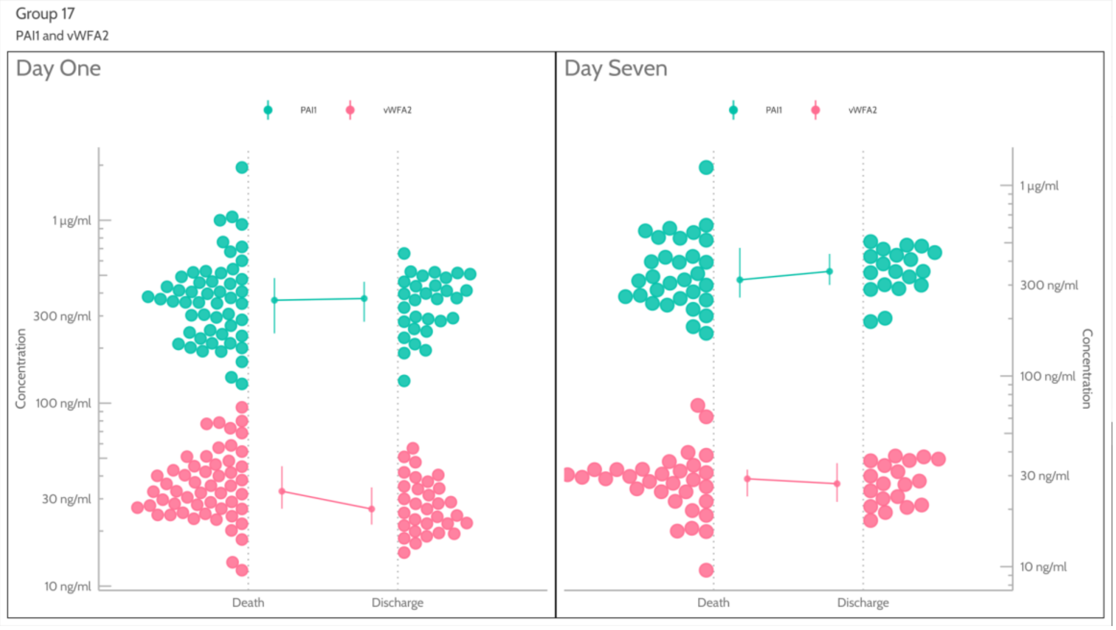
Group 17
PAI1 and vWFA2
Day One
PAI1
vWFA2
1 µg/ml
300 ng/ml
100 ng/ml
30 ng/ml
10 ng/ml
Concentration
Death
Discharge
Day Seven
PAI1
vWFA2
1 µg/ml
300 ng/ml
100 ng/ml
30 ng/ml
10 ng/ml
Concentration
Death
Discharge

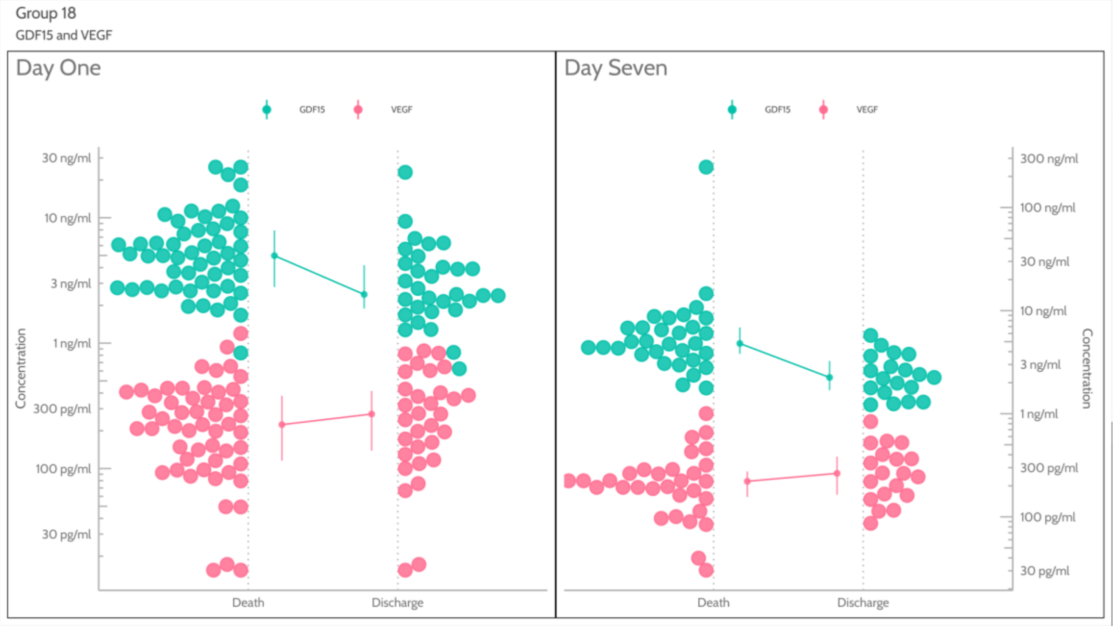
Group 18
GDF15 and VEGF
Day One
GDF15
VEGF
30 ng/ml
10 ng/ml
3 ng/ml
1 ng/ml
300 pg/ml
100 pg/ml
30 pg/ml
Concentration
Death
Discharge
Day Seven
GDF15
VEGF
300 ng/ml
100 ng/ml
30 ng/ml
10 ng/ml
3 ng/ml
1 ng/ml
300 pg/ml
100 pg/ml
30 pg/ml
Concentration
Death
Discharge

Supplementary Figure 3. Comparison of Day 1 and Day 7 biomarker panel performance metrics from the Random Forest and Logistic Regression models in the whole cohort compared to the HIV negative patients only

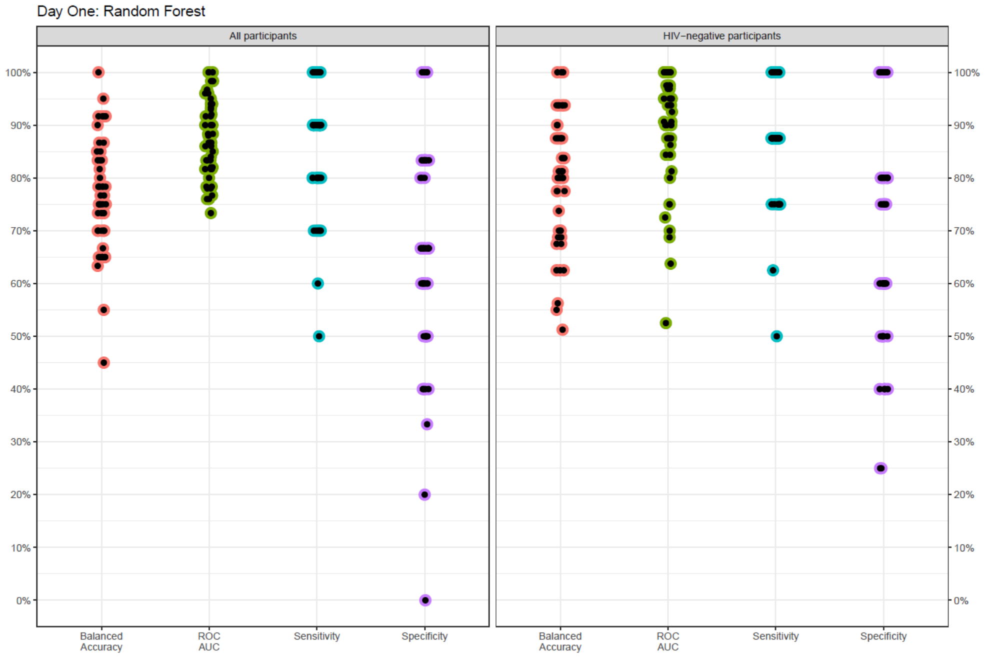

No significant differences in performance metrics were found between ‘all participants’ and ‘HIV negative participants’ on Welch's t-test.

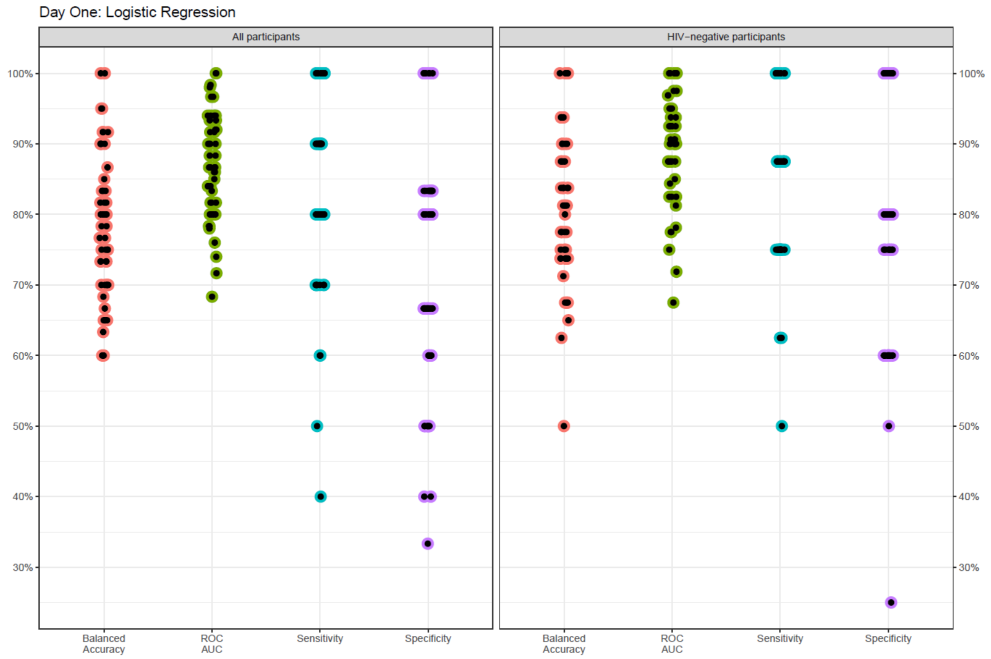

No significant differences in performance metrics were found between ‘all participants’ and ‘HIV negative participants’ on Welch's t-test.

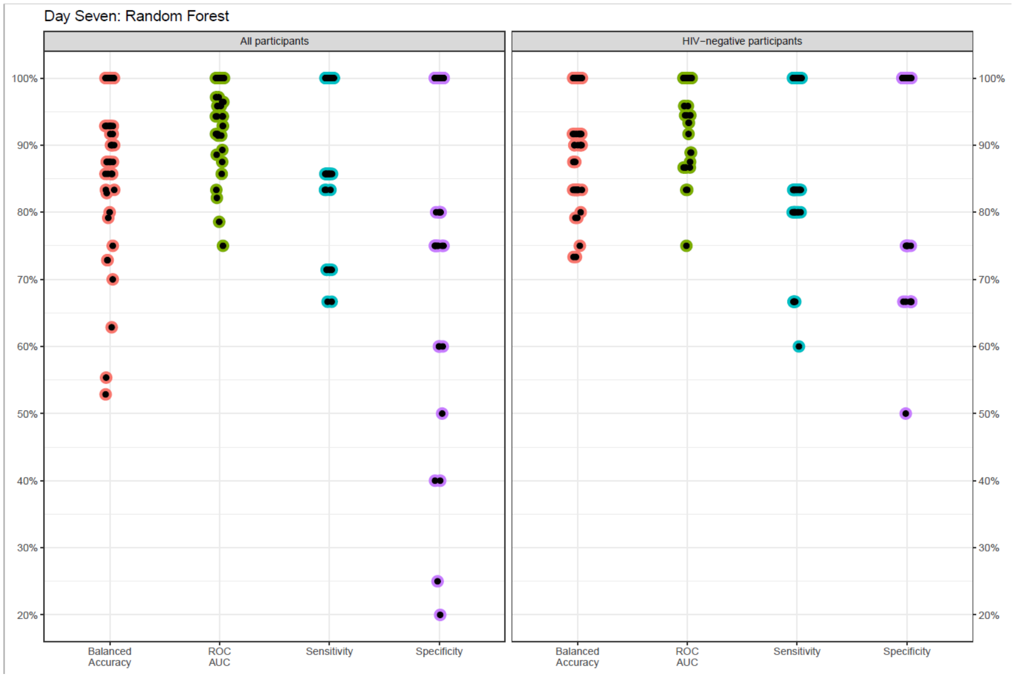

No significant differences in performance metrics were found between ‘all participants’ and ‘HIV negative participants’ on Welch's t-test.

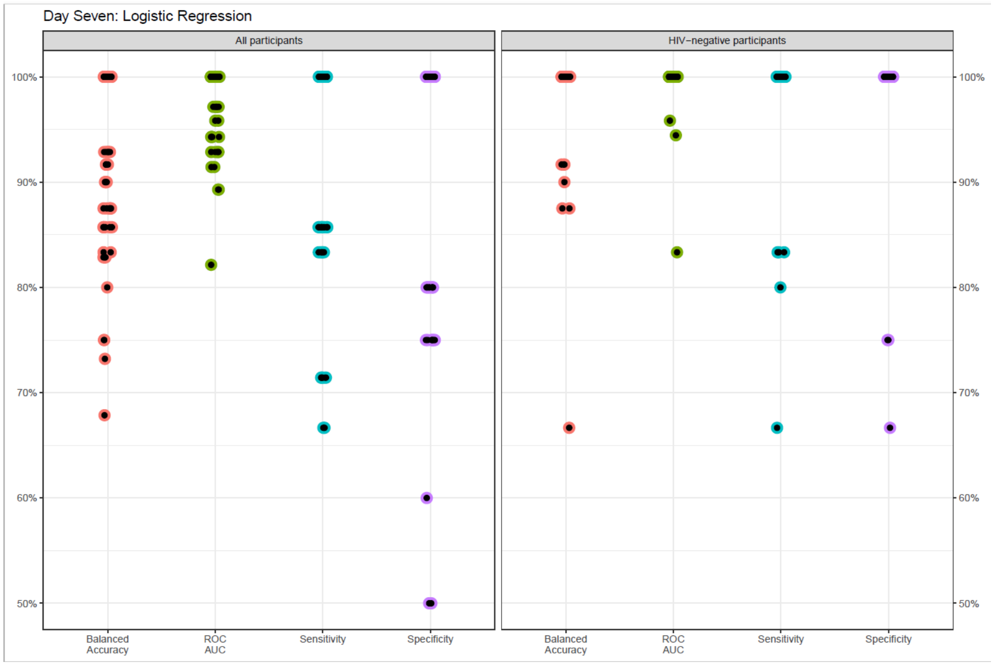

The ‘HIV-negative participants’ group performed better on metrics from logistic regression than the ‘all participants’ group when compared with Welch's t-test.

Supplementary Figure 4. Comparison of results of routine laboratory testing and Luminex® for C-reactive protein.

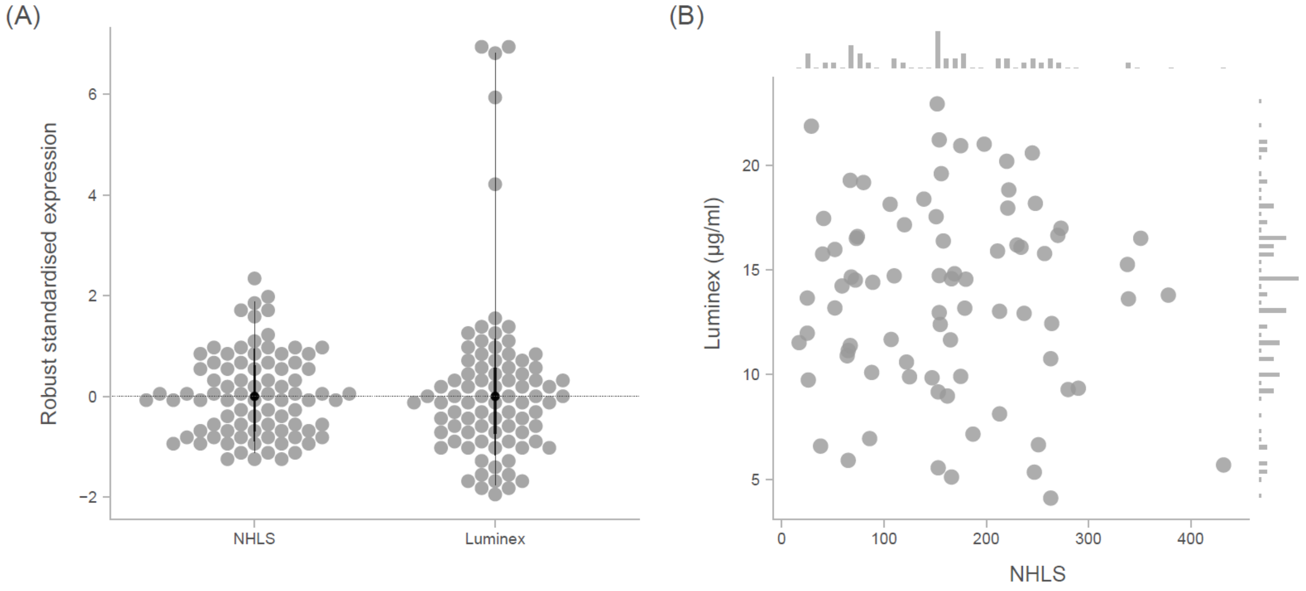

Panel (A) Assessment of distribution around the median after separate scaling and centering of values of C-reactive protein from the National Health Laboratory Services (NHLS, local reference laboratory) and Luminex® results. Panel (B) simple correlation plot. The NHLS laboratory that performs the routine blood tests uses a particle-enhanced immunoturbimetric assay (Tina-quant C-Reactive Protein IV, Roche Diagnostics GmbH, Mannheim) on the Roche Cobas® 6000 analyser (Basel, Switzerland) to determine the CRP. Higher baseline CRP from the NHLS platform correlated with mortality, but Luminex® CRP did not. This analysis shows that although the simple correlation plot does not find a correlation between the two assays, when the readings are individually centred and scaled, a robust t-test found the medians were not significantly dissimilar. This suggests that the assays still show the same trend even though one found a statistically significant association with the outcome and one did not.

Supplementary Figure 5. Comparison of results of routine laboratory testing and Luminex® for Procalcitonin.

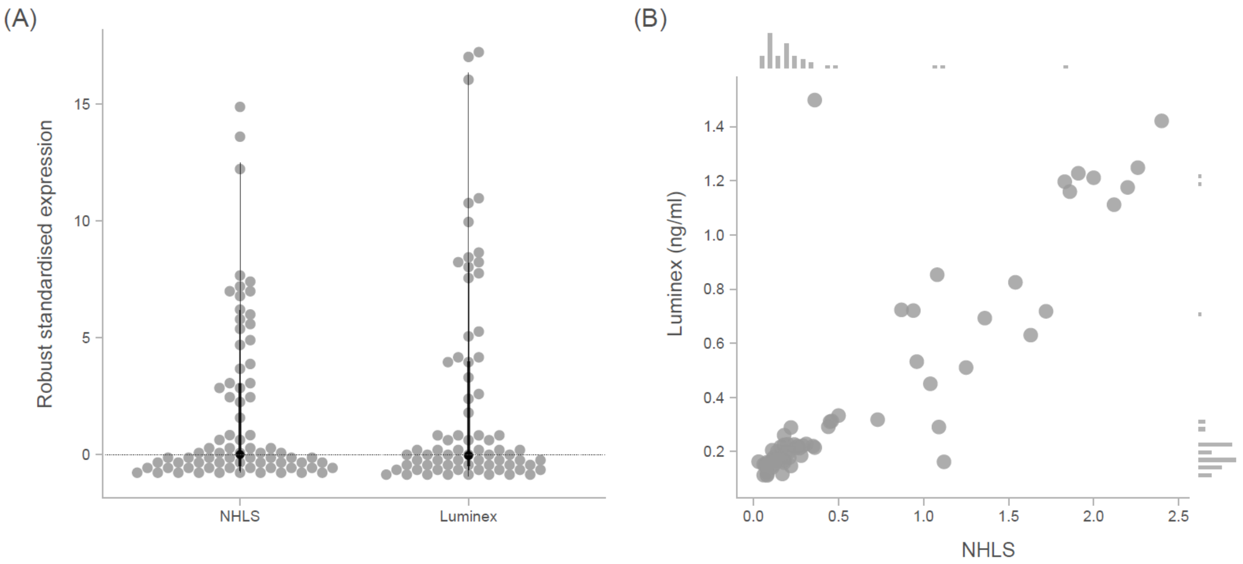

Panel (A) Assessment of distribution around the median after separate scaling and centering of values of procalcitonin from the National Health Laboratory Services (NHLS, local reference laboratory) and Luminex® results. Panel (B) simple correlation plot. The NHLS laboratory that performs the routine blood tests uses an electrochemiluminescent immunoassay (Elecsys BRAHMS PCT, Roche Diagnostics GmbH, Mannheim) on the Roche Cobas® 6000 analyser (Basel, Switzerland) to determine the PCT. Results for PCT from the NHLS platform and the Luminex® analysis correlated, and a robust t-test on centered scaled medians did not find a significant difference.
